# Tau seed amplification assay reveals relationship between seeding and pathological forms of tau in Alzheimer’s disease brain

**DOI:** 10.1186/s40478-023-01676-w

**Published:** 2023-11-14

**Authors:** Bryan Frey, David Holzinger, Keenan Taylor, Dagmar E. Ehrnhoefer, Andreas Striebinger, Sandra Biesinger, Laura Gasparini, Michael J. O’Neill, Florian Wegner, Stefan Barghorn, Günter U. Höglinger, Roland G. Heym

**Affiliations:** 1 AbbVie Deutschland GmbH & Co. KG, Neuroscience Research, Knollstrasse, 67061 Ludwigshafen, Germany; 2 AbbVie Bioresearch Center, Biotherapeutics and Genetic Medicine Technologies, Worcester, MA USA; 3 Department of Neurology, Hannover Medical School, Hanover, Germany; 4 Center for Systems Neuroscience, Hannover, Germany; 5 German Center for Neurodegenerative Diseases E.V. (DZNE), Munich, Germany; 6 Department of Neurology, LMU University Hospital, Ludwig-Maximilians-University (LMU), Munich, Germany

**Keywords:** Alzheimer’s disease, Tau seeding, Seed amplification assay (SAA), Real-time quaking induced conversion assay (RT-QuIC)

## Abstract

Tau seed amplification assays (SAAs) directly measure the seeding activity of tau and would therefore be ideal biomarkers for clinical trials targeting seeding-competent tau in Alzheimer’s disease (AD). However, the precise relationship between tau seeding measured by SAA and the levels of pathological forms of tau in the AD brain remains unknown. We developed a new tau SAA based on full-length 0N3R tau with sensitivity in the low fg/ml range and used it to characterize 103 brain samples from three independent cohorts. Tau seeding clearly discriminated between AD and control brain samples. Interestingly, seeding was absent in Progressive Supranuclear Palsy (PSP) putamen, suggesting that our tau SAA did not amplify 4R tau aggregates from PSP brain. The specificity of our tau SAA for AD brain was further supported by analysis of matched hippocampus and cerebellum samples. While seeding was detected in hippocampus from Braak stages I-II, no seeding was present in AD cerebellum that is devoid of tau inclusions. Analysis of 40 middle frontal gyrus samples encompassing all Braak stages showed that tau SAA seeding activity gradually increased with Braak stage. This relationship between seeding activity and the presence of tau inclusions in AD brain was further supported by robust correlations between tau SAA results and the levels of phosphorylated tau212/214, phosphorylated tau181, aggregated tau, and sarkosyl-insoluble tau. Strikingly, we detected tau seeding in the middle frontal gyrus already at Braak stage II-III, suggesting that tau SAA can detect tau pathology earlier than conventional immunohistochemical staining. In conclusion, our data suggest a quantitative relationship between tau seeding activity and pathological forms of tau in the human brain and provides an important basis for further development of tau SAA for accessible human samples.

**Supplementary Information:**

The online version contains supplementary material available at 10.1186/s40478-023-01676-w.

## Introduction

The widespread presence of tau neurofibrillary tangles (NFTs) in the brain is a hallmark of Alzheimer’s disease (AD) [1]. Such aggregated tau has been shown to act as a prion-like seed, causing misfolding and aggregation of physiological tau [2]. In addition, cell-to-cell transmission of tau seeds followed by seeded aggregation in healthy cells is considered to drive spatio-temporal propagation of tau pathology in the AD brain and contribute to the progression of the disease [3].

Consequently, several AD clinical trials are investigating attempts to slow tau propagation by targeting different mechanisms involved in tau seeding and aggregation [4]. A number of monoclonal antibodies in clinical trials target the mid-domain of tau [5, 6], which is directly involved in the formation of tau aggregates [7]. Other tau-targeted strategies in clinical trials include active vaccination, antisense oligonucleotides and aggregation inhibitors [8].

Established biomarker assays measure pathological forms of tau in living individuals [9]. For example, immunoassay quantification of phosphorylated tau (ptau) in biofluids [10] or detection of tau aggregates in the brain by positron emission tomography [11] enable classification of AD patients based on the underlying pathology. While currently available tau biomarkers are highly valuable for diagnosis and patient stratification, they do not directly monitor the prion-like behavior of tau. Direct measurement of tau seeding activity would therefore complement the current set of tau biomarkers, especially for investigation of target engagement and pharmacodynamic effects in clinical trials targeting tau seeds.

So far, seeding-competent tau species have been detected in post-mortem brain tissue by assessing the sample in cellular tau seeding assays [12–15]. In these assays, HEK cells overexpressing a short tau fragment fused to a fluorescent protein are incubated with brain homogenates. Tau seeding is then detected via intracellular fluorescent puncta indicating the formation of tau aggregates. In 2016, a study reported detection of tau seeding in one *ante mortem* AD CSF sample [16]. However, this result was not confirmed by a recent study using a tau cell seeding assay with improved analytical sensitivity [19]. It thus remains an open question if detection of tau seeding in AD biofluids by cellular assays is feasible.

We hypothesize that seed amplification assays (SAA), also known as real-time quaking-induced conversion assays (RT-QuIC), could fill this gap. The SAA method amplifies prion-like protein seeds in a biological sample via cycles of incubation and shaking in the presence of recombinant protein substrate and Thioflavin T [17]. The seeds induce aggregation of the protein substrate. The resulting aggregates are detected in real time by increased Thioflavin T fluorescence upon binding to amyloid structures in those aggregates. SAAs directly detect prion-like protein seeds with very high analytical sensitivity in the fg/ml range enabling detection of seeding in brain homogenates diluted down to 10^–9^ [17–20]. Since cell toxicity is not an issue, larger biofluid volumes can be used for SAAs, increasing the likelihood of detecting minute amounts of seeds [21]. Initially developed for prion protein [22, 23], the SAA technique is now also widely used to detect α-synuclein seeding in various biospecimens of Parkinson’s disease (PD) [24–30] and was recently proposed as an essential component of a biological definition of PD [30, 31].

To date, only three different groups reported studies on the use of SAA for detecting tau seeds [17–20, 32–34]. These include highly sensitive assays using short tau substrates with increased tendency to aggregate [17–20] and less sensitive assays applying full-length tau isoforms as substrates [33, 34]. Two studies reported selectivity for either 3R tau aggregates in Pick’s disease brain samples [17] or 4R tau aggregates in Progressive Supranuclear Palsy (PSP) brain samples [20]. This was achieved by using matching tau isoforms as substrates, i.e., a short 3R tau substrate for Pick’s disease and a short 4R tau substrate for PSP. Initial characterization of tau seeding signal in a small study of eight AD brains showed a relationship between tau seeding and Braak staging [18]. Very recently this has also been studied in a larger cohort with 16 AD brains, 35 brains with mixed neuropathologies such as PD or Multiple System Atrophy (MSA) and 12 control brains [35]. However, the precise relationship between tau seeding measured by SAA and the levels of AD-associated forms of tau, such as ptau, aggregated tau, or sarkosyl-insoluble tau remains unknown.

**Fig. 1:**
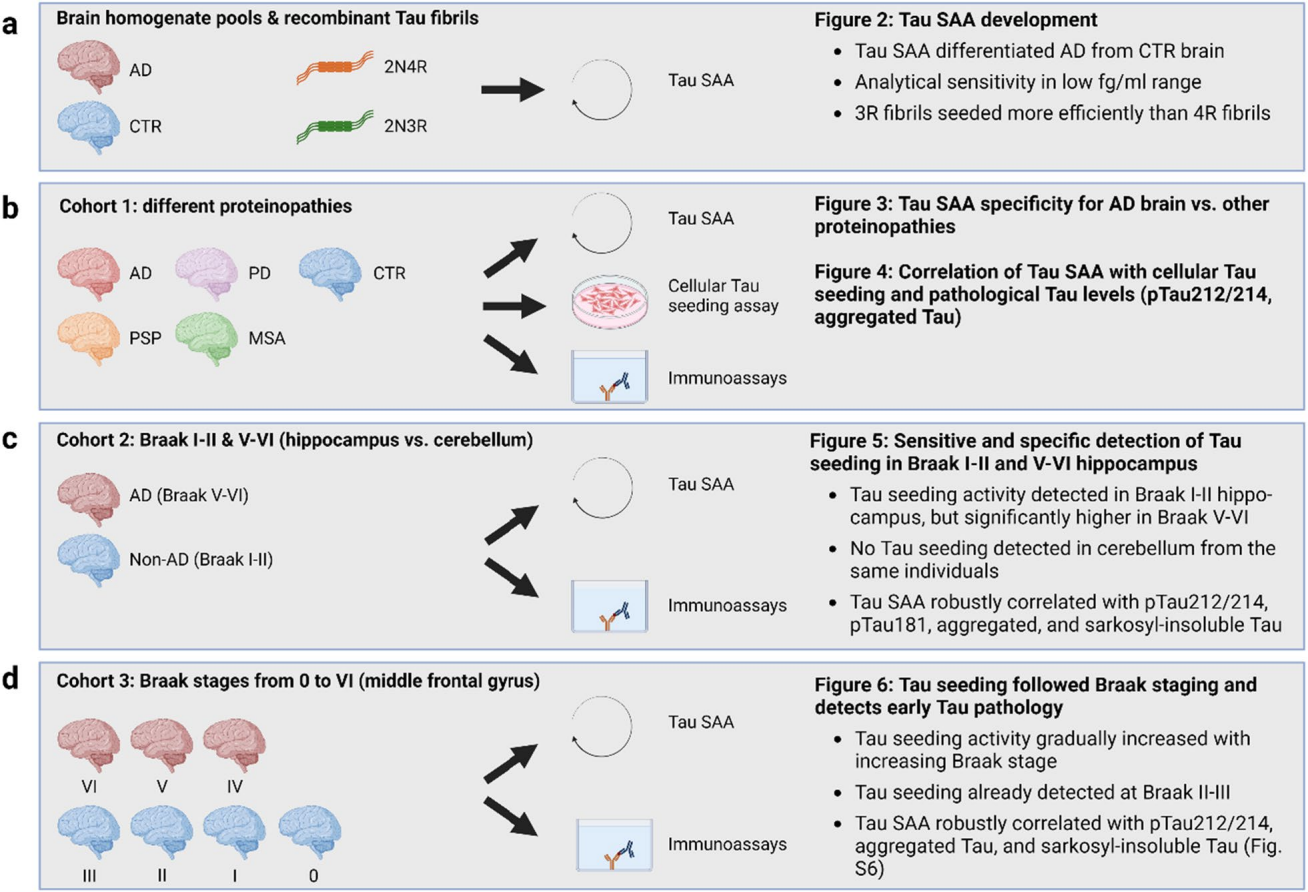
Overview of study design and main results. **a** Fig. 2. **b** Figs. 3 and 4. **c** Fig. 5. **d** Fig. 6. Roman numbers (I-VI) refer to the neuropathologically defined Braak stage. 2N4R and 2N3R refer to the Tau isoform used as recombinant Tau fibrils (Fig. 2). Tau SAA: Tau seed amplification assay, *AD* Alzheimer’s disease, *CTR* controls, *PSP* Progressive Supranuclear Palsy, *PD* Parkinson’s disease, *MSA* Multiple System Atrophy

To address this open question, we developed and validated a new SAA for AD brain using full-length 0N3R tau as substrate. Using this assay, we characterized a total of 103 brain samples from 3 different autopsy cohorts (Fig. 1). The first cohort (N = 43), consisting of mixed pathologies and brain regions, served to validate the assay. The second cohort (N = 35) was used to investigate the specificity of tau seeding detection by comparing AD hippocampus, carrying tau aggregates already at early Braak stages, with AD cerebellum that is devoid of tau aggregates. The third cohort (N = 40) consisted of middle frontal gyrus from brains spanning all Braak stages and was used to study the relationship between seeding and tau NFT pathology. Our investigations contribute to the understanding of tau seeding in AD brain and provide an important basis for future optimization of tau SAA for the analysis of accessible AD biosamples to aid future clinical trials.

## Materials and methods

### Brain tissue samples and compliance with ethical standards

Anonymized post-mortem brain samples were obtained from the Netherlands Brain Bank (NBB), Netherlands Institute for Neuroscience, Amsterdam (open access: www.brainbank.nl), as well as from commercial sources (Additional file 1: Tables S2, S3, S4), including Tissue Solutions Ltd (Glasgow, UK), Analytical Biological Services Inc. (Delaware, USA), Banner sun health research institute (Arizona, USA), Discovery life sciences (Alabama, USA), Folio Biosciences (by now merged to discovery life sciences). All materials were collected from donors for whom a written informed consent for a brain autopsy and the use of the material and clinical information for research purposes had been obtained.

### Sarkosyl-insoluble tau preparation

Tissue extraction was carried out on ice or at 4 °C, unless stated otherwise. Frozen human brain tissue samples were thawed on ice and homogenized in lysate buffer (50 mM Tris, pH 7.4, 150 mM NaCl, 20 mM NaF, 1 mM Na_3_VO_4_, 0.5 mM MgSO_4_, cOmplete protease inhibitor with EDTA (Roche, #11,697,498,001) and PhosSTOP phosphatase inhibitor (Roche, #04906845001) at 5 μl/mg tissue using a cooled bead disruptor (FastPrep-24 5G System, MP Biomedicals). Resulting brain homogenates were frozen in liquid nitrogen and stored at − 80 °C. Sarkosyl-insoluble tau was extracted according to the protocol described in a previous publication [36]. In brief, 1.2 ml of brain homogenate was centrifuged at 27,000 × g for 20 min (min), after which the pellet was resuspended in 1.2 ml of 10 mM Tris/HCl pH 7.4, 1 mM EGTA, 0.8 M NaCl, 10% sucrose, cOmplete protease inhibitor and PhosSTOP phosphatase inhibitor using an Omni Bead Ruptor (Omni International, Inc) at room temperature (RT) and then spun at 27, 000 × g for 20 min at 4 °C. The supernatant was transferred to a new tube, sarkosyl added to a final concentration of 1%, and incubated for 1.5 h at RT with 750 rpm shaking. Subsequently, the sarkosyl solution was centrifuged at 150,000 × g for 45 min, the resulting pellet was washed once 200 μl TBS (25 mM Tris pH 7.4, 3 mM KCl, 140 mM NaCl) and resuspended in 120 μl TBS by vortexing and four sonication pulses (2 s each) with a probe sonicator (Sonoplus, Bandelin) at 35% amplitude. Finally, sarkosyl-insoluble tau extracts were flash-frozen in liquid nitrogen and stored at − 80 °C until further use.

### Generation of tau pre-formed fibrils

2N4R tau with the P301L mutation in PBS (Gibco, #14,190) with 1 mM DTT (Serva, #20,710.3) was incubated for 15 min at 55 °C and subsequently cooled for 10 min on ice. Heparin (Sigma Aldrich, #H3393) was then added to a final concentration of 40 µM and fibrilization allowed for 7 days while shaking with 800 rpm at 37 °C. Tau fibrils where then collected by centrifugation with 20,817 × g for 2.5 h at 4 °C and resuspension of the pellet in 125 µl of PBS. Homogenization was achieved by sonification in a ChIP sonic digital sonifier (Branson, custom build, Germany) set to pulse on/off at 10 s / 10 s at an intensity of 70% for 30 min at 4 °C. Protein concentration was finally determined by bicinchoninic acid assay (BCA) (Pierce, #23,225) analysis. The tau fibrils were snap-frozen in liquid nitrogen and stored at − 80 °C until further use.

### Tau seed amplification assay (SAA)

**Fig. 2:**
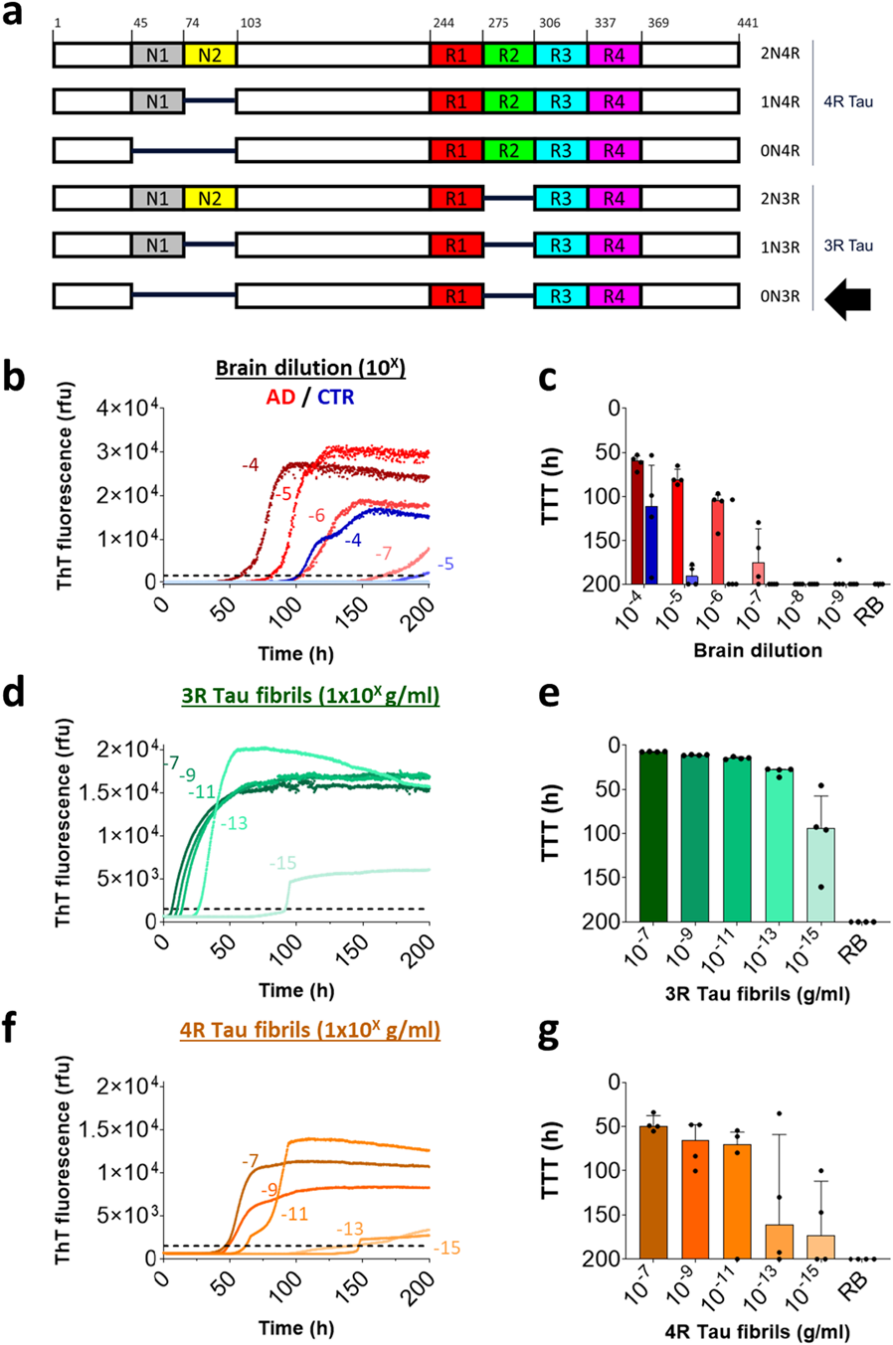
Tau SAA detected seeding by AD brain homogenate and recombinant Tau fibrils with very high sensitivity. **a** Illustration of the six Tau isoforms in the human brain with amino-terminal domains N1 and N2 and repeat domains R1-R4. Amino acid numbering on the top is referring to the 2N4R isoform. Arrow indicates the 0N3R Tau isoform used in Tau SAA. **b** Thioflavin T (ThT) kinetic curves of serially diluted AD (red) vs. control (CTR, blue) brain pools. Dotted lines depict the threshold of 1500 relative fluorescent units (rfu) for determination of the time to threshold (TTT) value. Curves are medians of technical quadruplicates. Numbering indicates dilution factor. **c** TTT values derived from curves shown in **b**. Each dot depicts a technical replicate, bars are medians, and error bars show the interquartile range. RB: reaction buffer. **d**–**e**. Tau SAA results of 2N3R Tau (green) fibrils. **f**–**g** Tau SAA results of 2N4R (orange) Tau fibrils

Full-length 0N3R tau (Fig. 2a) cysteine free (C322S) (SeNostic Health GmbH, Germany) in water, stored at a concentration of 1.0 mg/ml at − 80 °C, was thawed at RT and centrifuged with 21,000 × g at 4 °C for 30 min to remove potential preformed aggregates. The supernatant was adjusted to 0.1 mg/ml 0N3R tau in 40 mM HEPES pH 7.4, 400 mM NaF, 40 μM heparin (Sigma Aldrich, #H3393) and 10 μM Thioflavin T (ThT) (Sigma #T3516-25G), as reported by Metrick et al*.* [32]. After gentle mixing with the described SAA reaction buffer, 47 μl of the mixture was added to each well of a 384-well plate with an optically clear bottom (Greiner Bio-one, #781,906). Crude brain homogenates were thawed from − 80 °C storage at 16.7% (w/v) in PBS pH 7.4 supplemented with 1 × phosphatase inhibitors (Roche, #04906837001) and 1× protease inhibitors (Roche, # 04693124001) and serially diluted tenfold using a buffer containing 1 × N-2 Supplement (Gibco, #17,502–048), 10 mM HEPES and 0.53% crude brain homogenate from 8-month-old tau knockout mice (KO; B6.Cg-Mapt < tm1(EGFP) Klt > Tg(MAPT)8cPdav/J WT, Jackson Laboratories). The diluted mixture was then homogenized in TBS pH 7.4 with 1 × phosphatase inhibitors (Roche, #04906837001) and 1 × protease inhibitors (Roche, # 04693124001). Three µl of human brain homogenate dilutions were seeded into triplicates or quadruplicates in a reaction volume of 50 μl per well. We analyzed the seeding capacity in crude brain homogenates to preserve the integrity and activity of tau seeds and to avoid potential biochemical alterations, e.g. by extraction with detergents. Plates were sealed with fluorescence-permeable, clear adhesive sealing tape (Applied Biosystems, #4,311,971) and incubated in an Omega FLUOStar plate reader (bmg) set to 42 °C and with rounds of 1-min double orbital shaking at 500 rpm and 1 min resting, while fluorescence reads (450 excitation, 480 emission) were taken every 15 min. Please note that we did not observe evaporation due to leakage of the sealing tape during our assay runtime. Seed amplification kinetics were quantified using the time-to-threshold value (TTT) i.e., the time when the aggregation curve reached a ThT fluorescence threshold of 1500 relative fluorescence units (rfu). This threshold was empirically chosen aiming at sensitive detection of tau seeding activity while differentiating AD from non-AD cases. Lower TTT values indicate faster seeding kinetics due to higher levels of tau seeding activity in the biospecimen. To reflect the inverse relationship of TTT and seeding kinetics, we plotted the TTT values on an inverted axis throughout this study.

### Cellular tau seeding assay

HEK-293 T cells that stably expressed the tau microtubule-binding repeat domains (aa 244–342, numbering referred to 2N4R tau (Fig. 2a)) with the p301S mutation fused to yellow fluorescence protein (YFP) at their c-terminus were used as cellular tau seeding assay [2]. Stable cell lines, cultured in D-MEM (Gibco #31,966–047), 10% FBS and 100 µg/ml gentamicin at 8% CO_2_, were plated at a density of 2,000 cells/well in a black, poly-D-lysine coated 384 well plate (Corning # 354,663). At ~ 60% confluency, 24 h after plating, cells were transduced in quadruplicates with fibrillar tau seeds or human brain tissue that had been diluted in PBS with 0.1% Pluronic F68. These transduction complexes were made by combining Lipofectamine 2000 (Invitrogen #11,668–019) in Opti-MEM medium (Gibco #51,985–026) with the respective analyte dilution in a ratio of 1:1 for 10 min at RT. Subsequently, 20 µl liposome preparations were added directly to 20 µl cell culture medium per well and incubation allowed for 48 h. Next, cells were fixed and the nucleus stained by addition of 4% PFA (VWR #20,909.290), 4% sucrose (Sigma #S9378) and 2.5 µg/ml Hoechst-33342 (Thermo, #H3570) solved in DPBS in a final volume of 80 µl per well for 30 min at RT. Finally, fixed cells were washed three times with DPBS and stored at 4 °C until analysis in an array scan (Illumina) with an 20 × objective. Analysis was set up for 25 fields per well, exposure of 0.08 s and 25% target for Hoechst-33342 and 0.2 s with 40% target for the YFP measurement. Finally, tau-YFP spots were counted and the % of cells with spots determined by comparison to the number of Hoechst-33342-stained nuclei.

### Protein purification of hyperphosphorylated tau in Sf9 insect cells

Hyperphosphorytlated tau was generated and purified as described previously [37]. In brief, Sf9 cells were cultured in Sf900™ III SFM medium (Invitrogen, #10,658-027) with PenStrep (Invitrogen, #15,140-122) at 27.5 °C with a medium change every 4–5 days. Cells were diluted at 99% cell viability to 3–5 × 10^5^ cells/ml one day before transfection. On the next day, 1 µg bacmid DNA encoding for human 2N3R tau (pFastBac-huTAU (2N3R)) in 100 µl of Graces Insect Medium (Thermo, #11,595–030) were mixed with 2 µl Cellfectin II Reagent (Thermo, #10,362-010) in 100 µl Graces Insect Medium and incubated at RT for 30 min. An additional 800 µl of insect medium were added to the bacmid-Cellfectin mixture before mixing it with the Sf9 cells that were harvested via aspiration after a wash step with Graces Insect Medium. Transfection was allowed for 5 h at 27.5 °C. Cells were then incubated for 5 days in a 6-well plate with 2 ml of Sf900™ III SFM medium (Thermo, #10,658-027), PenStrep (Thermo, #15,140-122) and 10% of FCS at 27.5 °C. Successful expression of tau in the transfected Sf9 cells was verified by Western blotting, using tau5 (Thermo, #AHB0042) as primary antibody, anti-mouse-alkaline phosphatase conjugate (Promega, #S3728) as secondary antibody and Western Blue (Promega, #S3841) as alkaline phosphatase substrate. To purify the phosphorylated 2N3R tau, cell pellets were resuspended in lysis buffer (50 mM Tris–HCl [pH7.4], 500 mM NaCl, 10% glycerol, 0.1% Nonidet-P40, 10 mM EGTA, 20 mM NaF, 5 mM dithiothreitol, 1 mM orthovanadate) and lysed by sonication. The lysates were then centrifuged to eliminate cell debris, resuspended in lysis buffer and boiled for 10 min. This treatment ensures that nearly all proteins are denatured and precipitated, except for tau, which stays soluble. Precipitates were removed by centrifugation, the resulting supernatant was concentrated, loaded onto a Superdex 200 (GE Healthcare, #17-1071-01) and tau proteins were eluted in PBS, pH7.4, 1 mM DTT. Tau containing fractions were determined by SDS-PAGE and combined for dialysis against 100 mM MES pH6.8, 2 mM DTT, 1 mM NaEGTA, 1 mM MgSO4. Dialyzed samples were then subjected to ion exchange chromatography using a Q Sepharose column (GE Healthcare, #17-0510-01), where tau protein eluted with 0.2 M NaCl To facilitate buffer exchange into final formulation of PBS pH 7.4, 1 mM DTT, and to further purify the protein, an additional Superdex 200 chromatography step was performed. Finally, successful purification of ptau was ensured by i) immunblotting against total tau utilizing the tau5 antibody (Thermo Fisher, #AHB0042), as well as ptau utilizing the anti-ptau Thr-231 antibody (Thermo, # MN1040); ii) analytical size exclusion chromatography with a Superdex 200 and iii) mass spectrometry. More than 56% of purified tau displayed six to 11 phosphate groups while 44% displayed one to five phosphate groups, indicating a successful generation of hyperphosphorylated tau.

### ELISA

Total tau, aggregated tau, and ptau212/214 in human brain homogenates were quantified by ELISA, using tau-12 (Biolegend, #806,502), HT7 (Thermo, # MN1000), and AT100 (Thermo, #MN1060) as capture antibodies, respectively. Afterwards, biotin-HT7 (Thermo, # MN1000B) was utilized as detection antibody for each ELISA experiment. Tau 2N4R (Sigma T0576) was used as calibrator for the total tau ELISA. Pre-formed fibrils of 2N4R tau (P301L) were used as calibrator for the aggregated tau ELISA and hyperphosphorylated 2N3R tau for the ptau212/214 ELISA, each as described above. For plate coating with the respective capture antibody, 100 μl/well of 2 μg/ml antibody in PBS with 20% glycerol was adsorbed to Maxisorp NUNC-Immuno Plates (Thermo, #442,404) at 4 °C overnight. All following steps were performed at RT. The wells were washed three times with 250 µl PBST and blocked with 2% BSA (Serva #11,926) in PBST and 20% glycerol for 1.5 h. As a next step, all wells were washed three times with 250 µl PBST. Subsequently, calibrators (Total tau: 15.6–1000 pg/ml; aggregated tau: 78.13–5000 pg/ml; ptau212/214: 1562–100,000 pg/ml) and samples were diluted in PBST with 0.1% BSA and applied in duplicate. Plates were incubated for 2 h. After three additional washes of each well with 250 µl PBST, 100 µl of detection antibody biotin-HT7 at a concentration of 0.2 μg/ml was added and incubated for 1 h and subsequently washed again three times with 250 µl PBST. Next, 0.05 μg/ml Streptavidin Poly-HRP (Thermo, #21,140) in 0.1% BSA in PBST was added for 1 h, followed by another three washes with 250 µl PBST. Finally, 100 μl/well of TMB substrate (KEM-EN-TEC Diagnostics, #4800A) was added and the reaction stopped after 10 min by adding 100 μl 0.18 M sulphuric acid. The absorbance at 450 nm was then immediately measured in a Ledetect 96 plate reader (Anthos, Mikrosysteme GmbH). The lower limit of detection (LOD) was determined by the mean blank value plus two-fold standard deviation. A factor to be considered was that the phosphorylated tau (ptau) calibrator was not directly targeted to be phosphorylated at the AT100 sides, amino acids 212 and 214 (see above). Hence, only a portion of the total amount of ptau calibrator used to generate the standard curve was phosphorylated at the ptau 212/214 site. Consequently, ptau concentrations in the brain analytes were overestimated, when compared to e.g. total tau or aggregated tau ELISA concentrations.

### Singulex Erenna

Ptau181 capture mAb (Abcam, #ab236458) were conjugated to streptavidin Dynabeads following the Erenna Capture Kit manufacturer’s instructions (catalog #03–0077-02) to a final concentration of 0.25 mg/ml in bead buffer, provided in the kit. ptau standard (see section ELISA) (0.8–1000 pg/ml), blank and Brain samples diluted 1:25,000 or 1:50,000 in assay buffer (Merck #02–0474-00) were added to a 96-well plate (Axygen, #P-96-450V-C) in a final volume of 100 µl per well. Each analyte was then mixed with 100 µl of 0.05 mg/ml conjugated bead suspension in assay buffer (Merck #02–0474-00) and incubated for 2 h at RT on a shaking device (Boekel). After magnetic isolation, beads were washed with 1 × Erenna wash buffer (Merck, #02–0111-03) with a HydroFlex plate washer (Tecan Group AG). Subsequently, 20 µl of Alexa-647 fluorescently labeled tau12 detection antibody (500 ng/ml) (Biolegend, #806,502) were added to each well and the plate then placed on a Jitterbug shaker at RT for 1 h. After extensive washing, beads were eluted by incubation in 9.5 µl elution buffer (Merck 02–0297-00) for 20 min at RT on a Jitterbug shaker, transferred to a transparent 384-well plate (Thermo Fisher, #264,573) and the solution neutralized by addition of 10 µl/well neutralization buffer D (Merck #02-0368-00). Finally, the 384-well plate was heat-sealed with a variable temperature sealer (Thermo Fisher) and analyzed with the Singulex Erenna immunoassay system (Millipore). The LOD was defined as the lowest back interpolated standard and a coefficient of variance (CV) 20%.

### Statistical analysis

GraphPad Prism was used to perform statistical analyses. Differences among two groups were analyzed by Mann–Whitney test, while multiple comparison were performed by non-parametric one-way ANOVA Kruskal–Wallis test. A p-value of < 0.05 was considered statistically significant.

## Results

### Development of a sensitive tau SAA for AD brain

To identify a suitable substrate for SAA, we tested 11 different full-length tau constructs, including cysteine-free mutants to prevent intra- und inter-molecule disulfide bond formation which can prevent tau-fibril aggregation [38] (Additional file 1: Table S1). For the initial testing of all tau constructs, we used pooled AD and control brain homogenates to ensure assay optimization toward the detection of pathological tau species. Most tau constructs showed no aggregation under the tested conditions (Additional file 1: Table S1). 0N3R tau (Fig. 2a) with a cysteine-to-serine mutation (C322S) was the construct that most clearly discriminated AD from control brain, and did not show any self-aggregation in the absence of brain homogenate during a 200 h assay runtime (Additional file 1: Table S1). With increasing dilution of AD brain homogenate, we observed increasing time-to-threshold (TTT) values, i.e. later onset of aggregation, suggesting a quantitative relationship between the concentration of endogenous tau aggregates and seeding activity in tau SAA (Fig. 2b and c). Seeding by AD brain homogenate was detected down to a 10^–7^ dilution with ideal discrimination from control brain homogenate at a 10^−5^–10^–6^ dilution (Fig. 2b and c).

Tau NFTs in AD brain consist of a mixture of 3R and 4R tau [39]. To determine if the selected 0N3R tau substrate leads to preferential seeding by 3R vs. 4R tau, we tested serial dilutions of recombinant 2N3R and 2N4R tau fibrils (Fig. 2d–g). As for AD brain homogenate, we observed an increase in TTT with decreasing concentration of both 3R and 4R tau fibrils. Interestingly, TTT values were lower for 3R fibrils than for 4R fibrils, suggesting that seeding with 3R fibrils led to faster onset of aggregation. In agreement with the concept of templating [40], this result indicates that the 0N3R tau substrate is more compatible with seeding by 3R compared to 4R fibrils. Seeding by both tau fibrils could be detected down to concentrations of 1 fg/ml (equivalent of 2.2 × 10^–20^ M tau monomer), suggesting high sensitivity of our tau SAA.

Previous SAA studies with α-synuclein and prion protein showed that variabilities in substrate protein preparation can affect the performance of SAAs [41, 42]. Therefore, we tested two independent 0N3R (C322S) tau purification batches with 19 AD brain samples (Additional file 1: Table S2). Despite a tendency toward faster seeding kinetics of purification batch 2, we observed robust batch-to-batch correlation with a Spearman's r_s_ of 0.66 (Additional file 1: Fig. S1).

### Tau SAA discriminated AD from PSP, synucleinopathies and controls

In order to investigate the specificity of the tau SAA for AD as compared to other tauopathies and synucleinopathies, brain homogenates from 15 cases of AD, 3 cases of PSP, 3 cases of PD, 3 cases of MSA and 18 control cases were analyzed (cohort 1, Table 1 and Additional file 1: Table S2). The AD samples comprised hippocampus and different cortical regions demonstrating tau pathology at Braak stages V-VI. In addition, PSP putamen, PD substantia nigra, and MSA cerebellum were selected, since these regions carry high loads of tau and α-synuclein pathology, respectively. Matter from corresponding brain regions from individuals without neurodegenerative diseases were used as control samples.

**Table 1:** Basic characteristics of cohort 1

Disease	N	Age, mean and range (y)	Braak (Tau)	Sex (f/m)	Region	N	Figures
AD	15	69 (55–86)	V-VI	11/4	EC	2	3 and 4
					HIP	2	
					FC	5	
					TC	10	
PSP	3	80 (72–89)	n.d	1/2	PUT	3	
PD	3	80 (74–90)	0-II	0/3	SN	3	
MSA	3	61 (60–62)	n.d	3/0	CER	3	
CTR	19	74 (49–93)	I-II*	8/11	EC	4	
					HIP	3	
					TC	3	
					PUT	3	
					SN	3	
					CER	3	

Alzheimer’s disease (AD), Progressive supranuclear palsy (PSP), Parkinson’s disease (PD), Multiple system atrophy (MSA) and control (CTR) brain samples. Four AD patients donated two different brain areas (for details see Additional file 1: Table S1). Old, non-demented controls had neurofibrillary tangles classifying them as Braak I-II. EC: Entorhinal cortex; HIP: Hippocampus; FC: Frontal Cortex; TC: Temporal Cortex; PUT: Putamen; SN: Substantia Nigra; CER: Cerebellum; y: years; f: female; m: male; n.d.: not determined. *Indicated Braak stage (Tau) based on 3 C donors. For detailed description of cohort 1 samples, see Additional file 1: Table S2

**Fig. 3:**
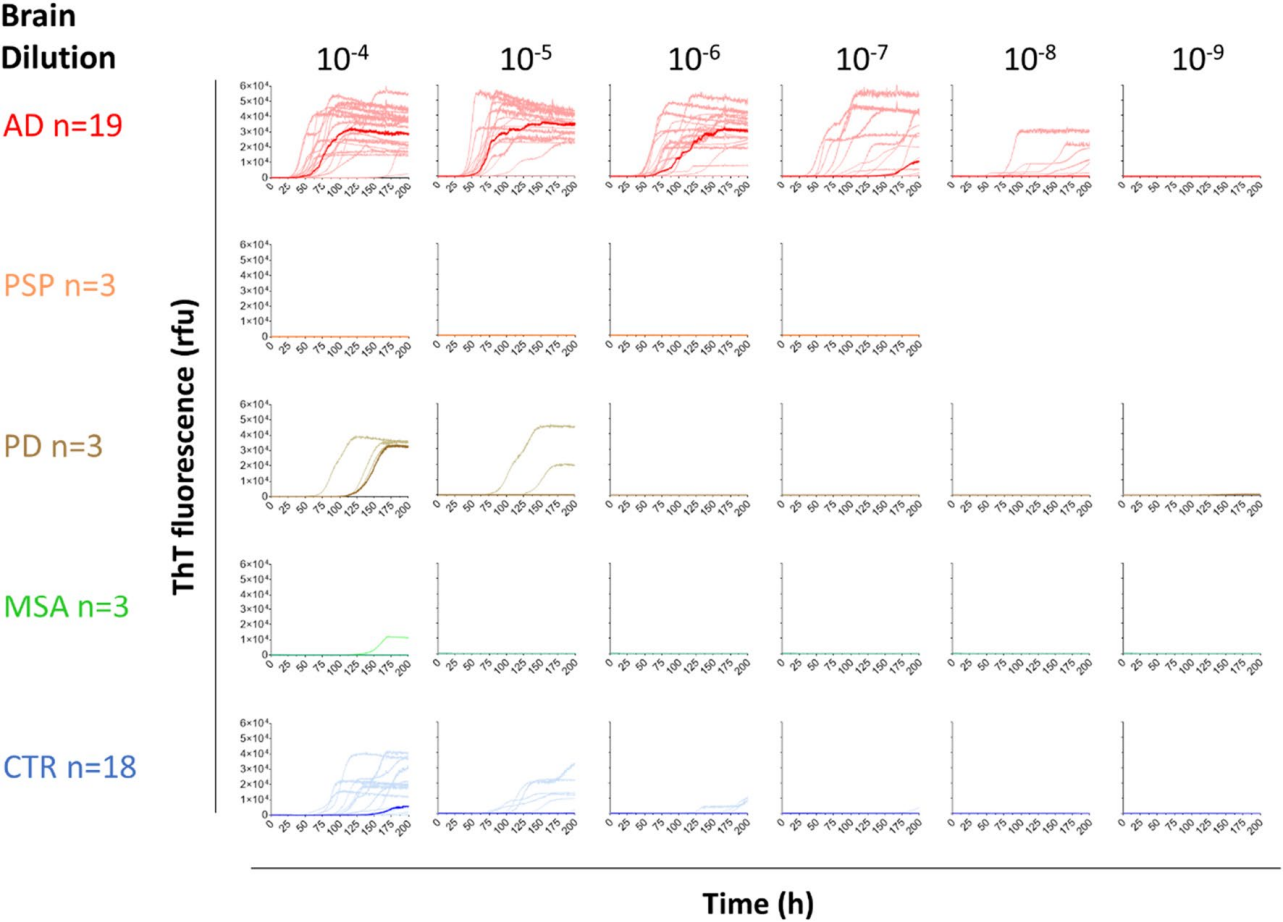
Tau SAA showed high specificity for AD compared to Progressive Supranuclear Palsy (PSP), Parkinson’s disease (PD), Multiple System Atrophy (MSA) and control (CTR) brains from cohort 1. Graphs show ThT kinetic curves of serially diluted AD (red, n = 19), PSP (orange, n = 3), PD (brown, n = 3), MSA (green, n = 3) and control (cyan, n = 18) brain homogenates. Dark colored curves depict the group median and light colored curves show the median of 3–4 technical replicates for each individual sample. Cohort 1 is described in detail in Table 1 and Additional file 1: Table S2

To identify the best dilution range for discrimination of AD from non-AD, brain homogenates were serially diluted, and tau SAA was monitored for 200 h. Once again, we observed a dose-dependent reduction in tau seeding activity with increasing dilution of AD brain homogenates (Fig. 3). At 10^–4^ dilution, some seeding was detected in samples from all groups except PSP, with the AD group showing faster seeding than all non-AD groups. At 10^–5^–10^–6^ dilution, fast seeding of the AD samples was observed, whereas the non-AD samples displayed no or much slower seeding. At 10^–7^–10^–8^ dilution, only a fraction of AD samples showed seeding, while no seeding was detected in any of the samples at 10^–9^ dilution. Seeding kinetics at a certain dilution level were similar across different AD brain regions (Additional file 1: Fig. S2). In summary, we conclude that a 10^–5^–10^–6^ dilution provides the best sensitivity and specificity for the detection of seeding in AD brain matter.

None of the tested PSP putamen samples showed seeding at the tested dilutions (Fig. 3). We therefore conclude that our tau SAA with 0N3R tau as substrate did not efficiently detect 4R tau inclusions present in PSP brain matter. Despite reports on cross-seeding of α-synuclein and tau [43, 44], neither PD nor MSA brain induced faster seeding than (nonpathological) control samples from the respective brain areas (Additional file 1: Fig. S2). It is therefore unlikely that cross-seeding by α-synuclein inclusions is a major confounding factor in our tau SAA. Interestingly, substantia nigra samples from two PD and two control cases showed slow seeding at 10^–5^ dilution (Additional file 1: Fig. S2). We also observed this in some of the hippocampus and cortex control samples, but not in putamen and cerebellum control samples (Additional file 1: Fig. S2). It indicates that different brain areas may generate different levels of background seeding signal in the tau SAA at 10^–5^ dilution, underlining the importance of comparing the same brain areas across disease and control groups.

### Tau SAA correlated with cellular tau seeding, phosphorylated tau and aggregated tau

**Fig. 4:**
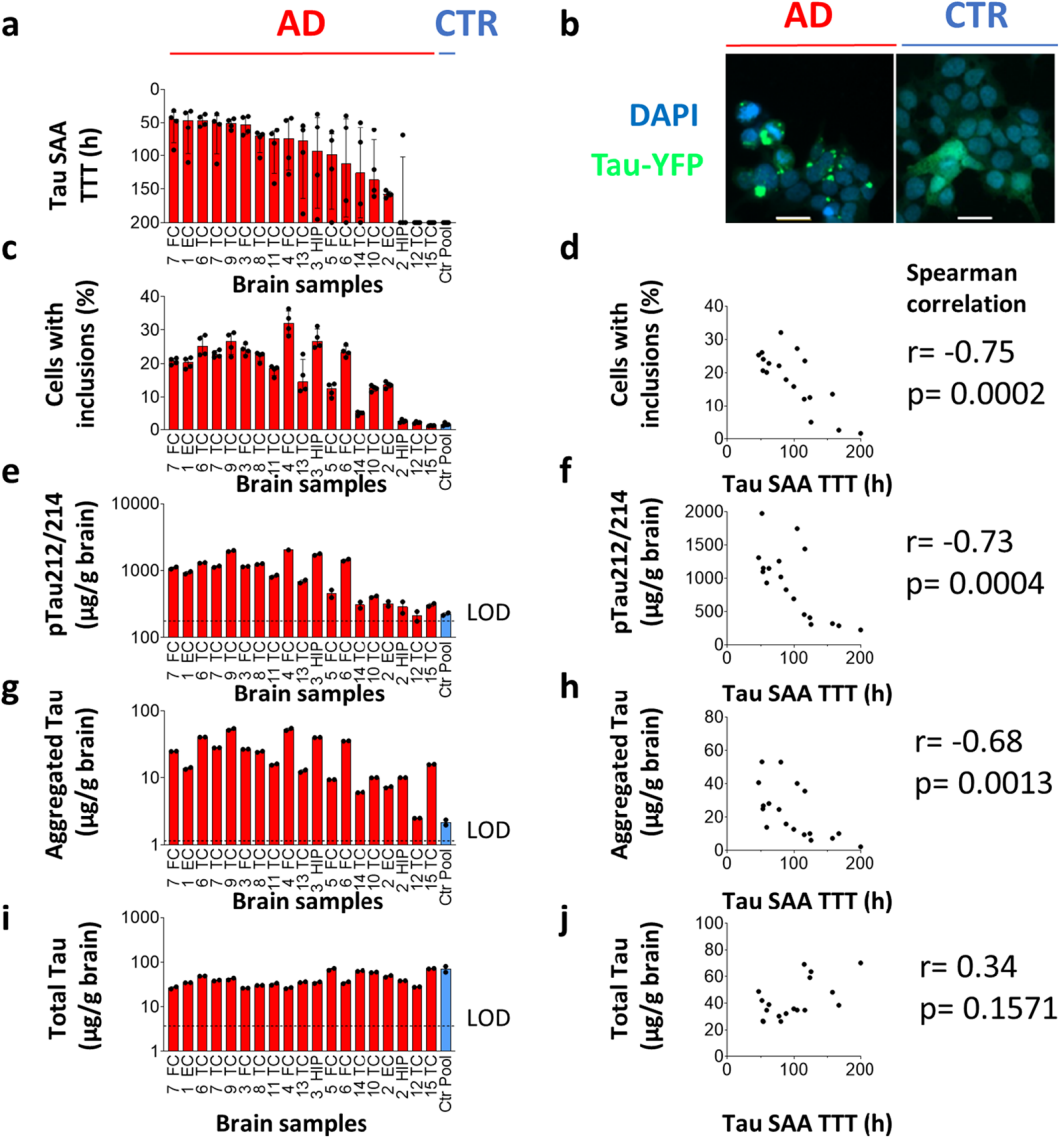
Tau SAA TTT of AD brain homogenates from cohort 1 correlated with Tau biosensor cell seeding assay, phosphorylated Tau, and aggregated Tau. **a** Nineteen AD brains (red) and one control brain pool (CTR, blue) were analyzed with Tau SAA. Tau SAA was performed with brain homogenates diluted 10^–6^. X-axis indicates case numbers and brain regions (EC: Entorhinal cortex; FC: Frontal Cortex; HIP: Hippocampus; TC: Temporal Cortex). Dots represent technical replicates. Bars and error bars indicate medians and interquartile ranges. **b** Representative images from Tau biosensor cell seeding assay. HEK293 cells stably expressing Tau repeat domains (amino acids 244–372) with P301S mutation fused to yellow fluorescent protein (Tau-YFP) were incubated with AD and CTR brain pools for 48h. Afterwards, cells were fixed, stained with DAPI and imaged for Tau-YFP (green) and DAPI (blue). Scale bar represents 15 µm. **c** Quantification of YFP-positive inclusions in Tau biosensor cell seeding assay incubated with brain homogenates diluted 1:8. **d** Spearman correlation of Tau SAA TTT with percent of cells with inclusions in Tau biosensor cell seeding assay. **e**–**j** Quantification of pTau212/214, aggregated Tau and total Tau by ELISA, and their Spearman correlation with Tau SAA TTT. Dotted lines show limits of detection (LOD). Cohort 1 is described in detail in Table 1 and Additional file 1: Table S2

Cellular assays are well established for the detection of tau seeding in AD brain samples [12–15]. We next compared all tau SAA results of the 19 AD brain samples from cohort 1 (Additional file 1: Table S2) (Fig. 4a) with an established tau biosensor cell seeding assay. In this assay, HEK293 cells showed stable expression of the 4R form of the microtubule binding region with P301S mutation fused to yellow fluorescent protein (Fig. 4b and c) [2]. In addition, we analyzed one control brain pool serving as non-seeding control. The percentage of cells with tau inclusions were negatively correlated with tau SAA TTT (Fig. 4d), confirming TTT as a robust readout for quantification of tau seeding activity in AD brains. Cellular tau seeding was measured at eightfold dilution of AD brain matter (Fig. 4b and c), while 216-fold dilution largely led to a loss of signal (data not shown). In contrast, tau SAA detected seeding in nearly all AD cases at 10^–6^ dilution (Fig. 4a) and was thus at least 4 × more sensitive than cellular tau seeding.

The presence of aggregated and phosphorylated forms of tau is a pathological hallmark of AD brains [45]. We therefore quantified ptau212/214 (tau phosphorylated at T212/S214), aggregated tau, and total tau, by ELISA in the same sample set (Fig. 4e and f). We chose ptau212/214 since it is one of the best ptau epitopes that differentiates between AD and control brains (Fig. 4e and f) [46]. Aggregated tau was quantified by a symmetrical ELISA using the HT7 antibody for capturing and detection, as described by Dujardin et al*.* (Fig. 4g and h) [14]. Since the epitope for HT7 is occupied after capturing, signal detection with the same antibody can only generate signals for dimeric, aggregated or higher aggregated forms of tau, but not for monomeric tau. Different tau calibrator proteins were used for total-, aggregated and phosphorylated tau ELISAs (see Materials & Methods). While this precludes direct comparison of absolute concentrations between these analytes, relative comparisons between samples are valid for a given analyte.

The levels of aggregated tau and ptau212/214 varied up to 10 to 30-fold between individual AD brain samples (Fig. 4e, g). In contrast, the levels of total tau were more comparable (Fig. 4i). While tau SAA TTT did not correlate with total tau (Fig. 4j), it did inversely correlate with ptau212/214 and aggregated tau (Fig. 4f, h). In summary, our results indicate that tau seeding activity as measured by tau SAA is related to the abundance of aggregated tau and ptau212/214 in AD brains.

### Differential tau seeding activity in Braak I-II and V-VI hippocampi reflected the levels of pathological tau forms

The load and distribution of tau aggregates in the AD brain is defined by Braak staging [47]. While the cerebellum remains devoid of tau aggregates in all Braak stages, the hippocampus is already affected from Braak stage II onwards [47]. We investigated tau seeding activity in hippocampi with high versus low tau burden. For this purpose, we analyzed hippocampi from a second cohort (cohort 2) comprising 11 AD brains at Braak stages V-VI and 10 Braak stages I-II (Table 2 and Additional file 1: Table S3). As negative control, we used donor-matched cerebella from 6 AD Braak stages V-VI and 8 Braak stages I-II.

**Table 2:** Basic characteristics of cohort 2

Disease	Region	N	Age mean and range (y)	Braak (Tau)	Sex (f/m)	Figure
AD	HIP	11	72 (58–84)	V-VI	6/5	5
AD	CER	6	72 (58–84)	V-VI	3/3	
Non-AD	HIP	10	86 (70–95)	I-II	6/4	
Non-AD	CER	8	87 (75–95)	I-II	5/3	

Hippocampus and cerebellum of AD (Braak V-VI) and non-AD (Braak I-II) donors including 6 AD and 8 non-AD cases with matched samples from the same donors. HIP: Hippocampus; CER: Cerebellum; AD: Alzheimer’s disease; y: years; f: female; m: male. For detailed description of cohort 2 samples, see Additional file 1: Table S3

**Fig. 5:**
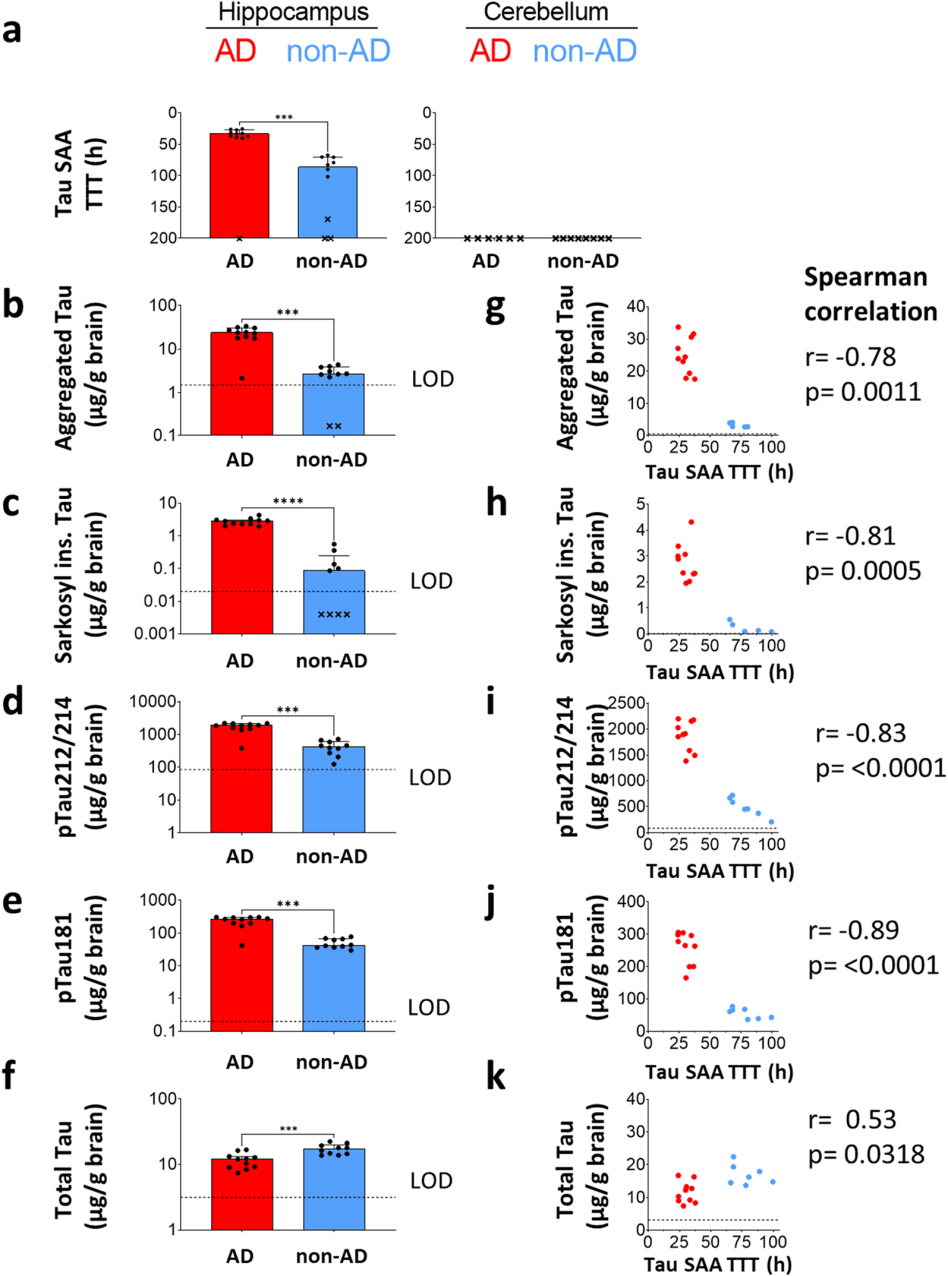
Sensitive detection of pathological and seeding-competent Tau forms in AD (Braak V-VI) and non-AD (Braak I-II) hippocampus. **a** Tau-SAA time to threshold (TTT) values of AD (Braak V-VI) and non-AD (Braak I-II) hippocampus (Hip) and cerebellum (Cer) homogenates (see Additional file 1: Table S3), diluted by 10^–6^. Dots indicate medians of technical quadruplicates per brain; crosses: non-seeding samples with at least 3 out of 4 technical replicates showing TTT values ≥ 200 h. **b**-**f** Immunoassay results for aggregated Tau, sarkosyl-insoluble Tau, pTau212/214, pTau181, and total Tau. Dots: mean of technical duplicates. Dotted lines indicate the limit of detection (LOD). Bars: group medians + interquartile range. Statistical comparisons of group medians were performed by Mann–Whitney test. *p < 0.05, **p < 0.01, ***p < 0.001. **g**–**k**. Spearman correlation of Tau-SAA TTT versus immunoassay results. Non-seeding samples and values below the LOD were excluded. Cohort 2 is described in detail in Table 2 and Additional file 1: Table S3

The AD hippocampi showed fast seeding with a median TTT of 33 h (Fig. 5a). Interestingly, seeding was also detected in hippocampi of Braak stage I-II cases, albeit with a significantly higher median TTT of 86 h. Notably, there was no overlap between TTT values of AD and Braak stages I-II groups except for a single AD case that did not seed within 200 h. None of the matching cerebella displayed tau seeding within 200 h (Fig. 5a), in agreement with the absence of tau inclusions according to the neuropathological reports (Additional file 1: Table S3).

To investigate the aforementioned relationship further, we quantified aggregated tau, ptau212/214, ptau181, and total tau. In addition, we utilized pre-existing data on sarkosyl-insoluble tau from this cohort [36]. Aggregated tau, sarkosyl-insoluble tau, ptau212/214, and ptau181 were all significantly elevated in AD versus Braak stages I-II hippocampi (Fig. 5b–e). In contrast, there was a significant reduction of total tau (Fig. 5f). This apparent reduction of total tau may stem from the high levels of aggregated tau in AD which could have caused steric hindrance of antibody binding and consequently lower signals in ELISA. Spearman analysis revealed robust inverse correlations of tau SAA TTT with all four pathological forms of tau, while a weak positive correlation was observed for total tau (Fig. 5g–k).

To better understand the relationship between tau seeding activity and pathological forms of tau in the brain, we stratified our immunoassay data based on tau SAA (Additional file 1: Fig. S3). One AD and three Braak stages I-II hippocampus samples did not show seeding within 200 h (Fig. 5a, crosses) and were thus grouped as seeding-negative. In agreement with tau SAA, the three seeding-negative Braak stages I-II hippocampi were devoid of tau NFTs according to the neuropathological reports (Additional file 1: Table S3) and did not carry detectable levels of sarkosyl-insoluble tau (Additional file 1: Fig. S3). In contrast, the one seeding-negative AD hippocampus carried many tau NFTs (Additional file 1: Table S3) and a high level of sarkosyl-insoluble tau, but the levels of aggregated tau, ptau212/214, and ptau181 were relatively low (Additional file 1: Fig. S3).

In summary, tau SAA seeding activity was significantly higher in AD Braak stages V-VI than in Braak I-II hippocampi, whereas no seeding was observed in matched cerebella devoid of tau NFTs. The detection of tau seeding in hippocampi from Braak I-II stage brains corresponded with the presence of pathological forms of tau measured by four different immunoassays and neuropathological assessment of tau NFTs. All four pathological forms of tau showed a robust correlation with tau SAA seeding activity.

### Tau seeding activity in middle frontal gyrus gradually increased with Braak stage reflecting the load of pathological tau forms

**Fig. 6:**
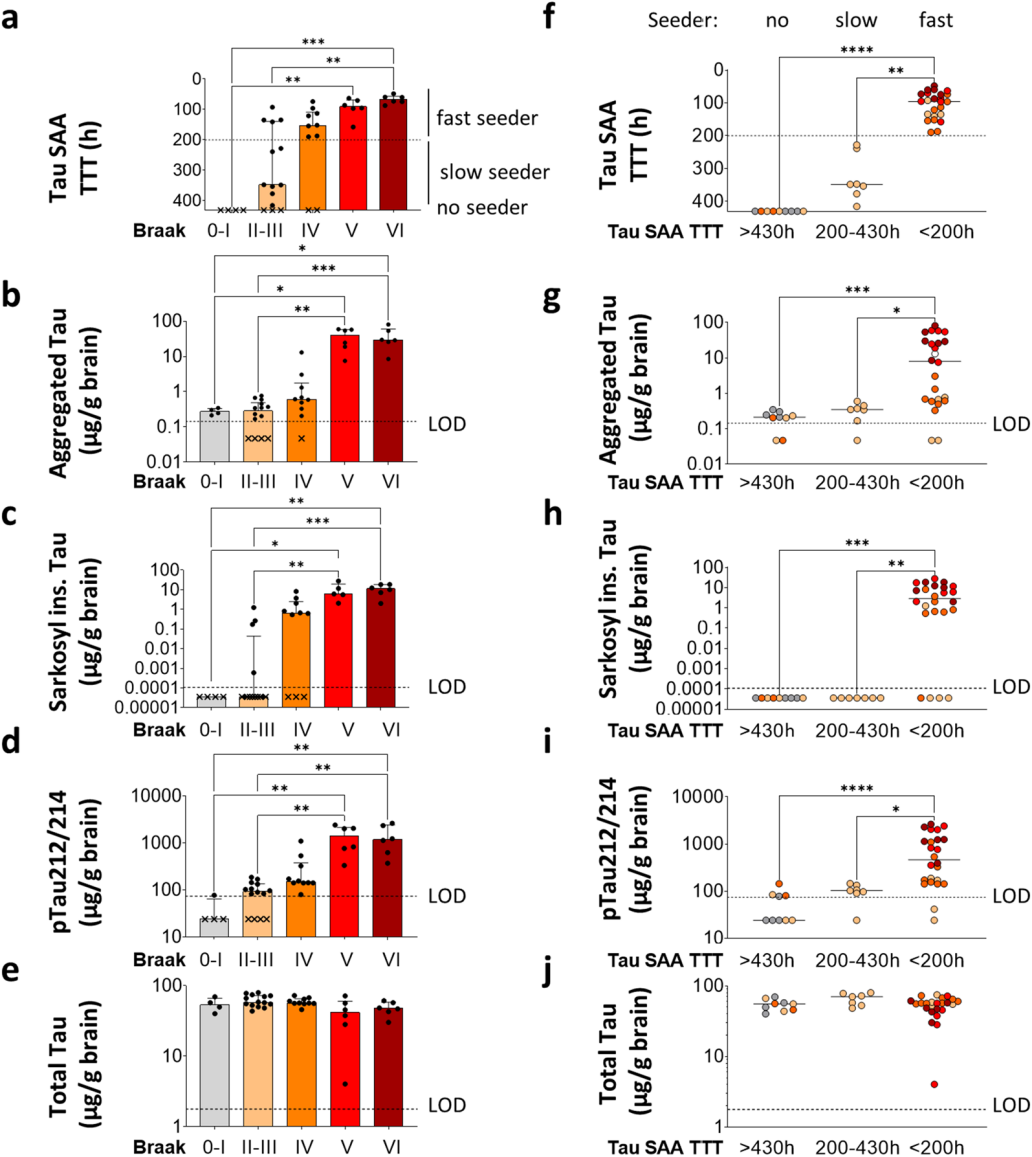
Tau-SAA seeding activity in middle frontal gyrus gradually increased with Braak stage (0-VI) and reflected the load of pathological Tau forms. **a** Time to threshold (TTT) values from Tau SAA of middle frontal gyrus homogenates (see Additional file 1: Table S4) diluted 10−5. Dotted line: separating fast-seeding (TTT < 200 h) and slow-seeding samples (TTT of 200–430 h). Crosses: non-seeding samples (TTT of ≥ 430 h). Dots depict the mean TTT values of technical triplicates. Bars represent the group medians + interquartile range. Statistical comparisons by Kruskal–Wallis test with Dunn’s multiple comparisons correction. *p < 0.05, **p < 0.01, ***p < 0.001. **b**–**e** ELISA results of aggregated Tau, sarkosyl-insoluble Tau, pTau212/214, and total Tau. Dots depict the mean of technical duplicates. Crosses in indicate values below the limit of detection (LOD, dotted line) and were set to one third of the LOD. **f**–**j** Stratification of samples into fast- (TTT <200 h), slow- (TTT of 200–430 h) and no seeding (TTT of ≥ 430 h) based on Tau SAA. Braak stages are colored as indicated in **a**–**e**. Vertical lines represent the group medians. Statistical comparisons by Kruskal–Wallis test with Dunn’s multiple comparisons correction. *p < 0.05, **p < 0.01, ***p < 0.001. Cohort 3 is described in detail in Table 3 and Additional file 1: Table S4

To further investigate the relationship between tau seeding and tau NFT load in the brain, we analyzed middle frontal gyrus samples from 40 AD and non-dementia control brains ranging from Braak stage 0 to VI (cohort 3, Table 3 and Additional file 1: Table S4). This brain area carries tau NFTs from Braak stage IV onwards according to neuropathological immunostaining [47]. In order to maximize detection of seeding, we recorded tau SAA over a prolonged time of 430 h and applied a brain homogenate dilution of 10^–5^. Under these conditions, we observed a gradual decrease of median TTT with increasing Braak stage: 0-I) > 430 h; II-III) 348 h; IV) 153 h; V) 91 h; VI) 66 h (Fig. 6a). Except for two samples, Braak IV-VI samples showed robust seeding with TTT < 200 h, confirming that TAU SAA results generally concur with the presence of TAU NFTs as detected by immunostaining. In contrast, Braak 0-I samples showed no seeding. Interestingly, most Braak II-III samples showed seeding with very variable kinetics (TTT ranging from 92 to 415 h). Similar variability was present in both the Braak II and the Braak III group when analyzed separately (Additional file 1: Fig. S5). These results indicate that prolonged recording over 430 h enables sensitive detection of seeding in Braak II-III middle frontal gyrus samples that do not yet contain immunostaining-detectable tau NFTs.

**Table 3:** Basic characteristics of cohort 3

Cases (Braak)	N	Sex (f/m)	Age, mean and range (y)	Figure
0—I	4	3/1	86 (78–93)	6
II—III	14	11/3	87 (76–99)	
IV	10	7/3	88 (72–100)	
V	6	3/3	75 (70–82)	
VI	6	4/2	70 (55–84)	

Middle frontal gyrus from 40 brains ranging from Braak 0 to VI

*y* Years; *f* Female; *m* Male. For detailed description of cohort 4 samples, see Additional file 1: Table S3

To characterize the relationship between tau seeding and pathological tau forms, we performed a set of tau ELISAs described previously for cohorts 1 and 2 (Fig. 6b-e). Soluble, total tau levels did not significantly differ between Braak stages (Fig. 6e). In contrast, levels of sarkosyl-insoluble tau, aggregated tau, and ptau212/214 were all significantly higher in Braak V and VI than in Braak 0-I and II-III samples (Fig. 6b–d), mirroring the tau SAA results (Fig. 6a). In line with the results from cohorts 1 and 2, tau SAA TTT correlated inversely with aggregated tau, sarkosyl-insoluble tau and ptau212/214, but not with total tau (Additional file 1: Fig. S6).

To better understand the relationship between tau SAA and pathological tau forms, we stratified our data into three categories based on tau SAA seeding kinetics (Fig. 6f–j): fast seeding (TTT < 200 h), slow seeding (200 h ≤ TTT < 430 h), and no seeding (TTT ≥ 430 h). The fast-seeding group showed significantly elevated levels of aggregated tau (Fig. 6 g), sarkosyl-insoluble tau (Fig. 6h), and ptau212/214 (Fig. 6i) as compared to non-seeding and slow-seeding groups. No sarkosyl-insoluble tau was detected in the slow-seeding group (Fig. 6 h). Since this group consisted entirely of Braak II-III samples, this finding is concurs with the absence of tau NFT immunostaining in the middle frontal gyrus. Interestingly, low levels of aggregated tau (Fig. 6 g) and ptau212/214 (Fig. 6i) were detected in the slow-seeding group.

## Discussion

We performed an in-depth characterization of tau seeding in human brains using a newly developed full-length tau SAA and compared it to the levels of different pathological forms of tau in the same samples. We analyzed three autopsy cohorts comprising 103 brain samples with neuropathological diagnoses of AD, PSP, PD, MSA, and matched controls (Fig. 1). To the best of our knowledge, this is the largest cohort studied by tau SAA to date and the first head-to-head comparison with multiple forms of pathological tau in AD brain.

In summary, our data showed high sensitivity and specificity of tau SAA for AD versus PSP, PD, MSA and controls without proteinopathies. Tau seeding activity in AD brain increased in concordance with tau NFT pathology classified by Braak staging and was significantly correlated with the levels of ptau181, ptau212/214, ptau231, aggregated tau and sarkosyl-insoluble tau. Our results on hippocampus and middle frontal gyrus from brains with Braak stages I-III indicate that tau seeding, ptau, and aggregated tau are earlier markers of AD pathology, while sarkosyl-insoluble tau was detected only at later Braak stages.

### Full-length 0N3R tau shows highest sensitivity and specificity among SAA substrates for AD brain

By comparing different full-length 3R and 4R tau constructs (Additional file 1: Table S1), we identified 0N3R tau with C322S mutation as the best substrate for amplification of tau seeds from AD brain. Most published tau SAAs are based on short tau constructs as substrates [17–20, 32, 48]. Only two previous studies used full-length tau substrates but studies reported limited sensitivity and specificity for AD brain [33, 34]. In contrast, our newly developed full-length tau SAA displayed high sensitivity for AD brain samples. The identification of 0N3R tau as suitable substrate for tau seeding is in agreement with previous studies on the aggregation of different tau isoforms. 0N tau isoforms were shown to be enriched in the insoluble fraction of AD brain, leading the authors to conclude that this isoform may be predisposed to aggregate [46]. It was also shown that 3R tau is more prone to form oligomers compared to 4R tau [49].

The use of different tau substrates could lead to preferential amplification of certain seeding-competent forms of tau and ultimately to deviations in SAA results. For example, it has been shown that aggregated 4R tau fibrils incorporate only 4R tau, while 3R fibrils can incorporate both tau isoforms [40]. Interestingly, a previous SAA study using a mixture of two artificial short tau substrates unexpectedly detected a low level of tau seeding in AD cerebellum, a region that does not display tau aggregates by classical immunostaining [18]. It is noteworthy that both cerebellum donors were also diagnosed with cerebral amyloid angiopathy in addition to AD. In contrast, in our study tau seeding was undetectable in AD cerebellum with full-length tau SAA (Fig. 5). Comparative studies on the same sample sets would be needed to further investigate similarities and differences between existing tau SAAs.

### Tau SAA selectively detected seeding in AD vs. PSP, PD, and MSA brain

Previous cell-based studies indicate that cross-seeding between tau and α-synuclein is generally possible [43, 44]. Therefore, we investigated brain samples from PD and MSA carrying Lewy bodies/neurites and glial cytoplasmic inclusions, respectively. We found that AD cortex and hippocampus exhibited orders of magnitude faster tau seeding, compared to PD putamen and MSA cerebellum which behaved similarly to control brain samples (Fig. 3 and Additional file 1: S2). This result indicated that α-synuclein seeds from PD or MSA brain did not efficiently cross-seed 0N3R tau. We conclude that potential α-synuclein co-pathology in AD brain is unlikely to impact the measurement of seeding by tau SAA.

Different tauopathies can be distinguished by the tau isoforms that form aggregates resulting in distinct fibrillar structures [50]. Aggregated 4R tau is found in PSP brain [50], while 3R tau aggregates are characteristic for Pick’s disease brain [51] and tau aggregates composed of both 3R and 4R tau are typical for AD brain [52, 53]. To investigate whether the 3R tau substrate of our SAA would distinguish 4R tau aggregates in PSP from 3R/4R tau aggregates in AD, we evaluated PSP putamen, the area with the highest tau aggregate load in PSP brain [54, 55]. The presence of tau inclusions in the tested PSP brain samples was verified by detection of phosphorylated tau via AT8 and AT100 immunohistochemical staining (data not shown). In contrast to AD, we did not detect tau seeding in PSP with our 3R tau SAA (Fig. 3, Additional file 1: S2-3). It cannot be ruled out that the lower abundance of tau inclusions in PSP compared to AD brain [56] contributes to this observation. However, it is likely that the 3R tau substrate in our SAA does not efficiently amplify 4R tau aggregates from PSP brain under the applied reaction conditions. This hypothesis is supported by the lower sensitivity of our tau SAA for recombinant 4R tau versus 3R tau fibrils (Fig. 2). In addition, it concurs with a previously reported tau SAA based on a short 3R tau substrate that detected strongly reduced seeding in PSP as compared to AD brain [32].

### Tau seeding activity was detected in middle frontal gyrus without tau NFT pathology

It is well established that the spatial distribution of tau NFTs described by the Braak staging is correlated with the degree of cognitive impairment in AD [57]. While tau NFT immunostaining can be found in the hippocampus of non-AD brains with Braak stage I-III [13], no tau inclusions are present in the frontal lobe at these stages [1, 58]. Detection of tau seeding in the hippocampus of Braak I-II cases (Fig. 5a) is thus in agreement with the presence of tau NFTs. As expected, tau seeding activity was significantly higher in AD (Braak V-VI) as compared to non-AD (Braak I-II) hippocampus. Interestingly, we also detected tau seeding in the middle frontal gyrus from Braak II-III cases (Fig. 6a), indicating that seeding-competent tau can be detected by SAA before tau NFTs appear in immunostaining. It is worth noting that a longer run time of the assay enabled detection of tau seeding in middle frontal gyrus of non-AD (Braak II-III) cases. Therefore, longer run times may be considered to increase SAA sensitivity, provided that adequate control cases are analyzed in parallel to exclude false-positive signals due to self-aggregation of the tau substrate.

### Tau seeding activity is related to the abundance of ptau212/214, ptau181, and aggregated tau in AD brain

Despite six tau SAAs studies published to date [17–20, 33, 34], the relationship between tau seeding activity and the levels of different pathological forms of tau in the brain remains unclear. We measured total tau as a control that is not directly relevant for seeding [15]. Total tau levels were significantly lower in AD vs. non-AD hippocampus from cohort 2 (Fig. 5) but did not differ significantly between Braak stages in cohort 3 (Fig. 6). We interpret this finding with caution: due to steric hindrance aggregated tau can be less accessible for pan-tau antibodies in sandwich ELISA as compared to monomeric tau. It is therefore likely that our total tau ELISA underestimated the concentration of total tau in AD brain regions with high levels of tau aggregates, such as AD hippocampus.

Phospho-tau212/214 showed a robust correlation with tau seeding in all three cohorts that comprised different brain areas across all Braak stages (Figs. 4, 5 and Additional file 1: Fig. S6). This finding is in line with a prior study by Wesseling et al. that demonstrated that seeding-competent high molecular weight tau oligomers were ptau212/214 positive [46]. In line with these findings, we observed that ptau212/214 correlated well with tau seeding in hippocampus of Braak stage I-II cases (Fig. 5i, blue dots, Spearman's r_s_ = -0.93, p = 0.007). Phospho-tau181 was significantly elevated in Braak V-VI versus Braak 1-II hippocampus and showed a robust correlation with tau seeding (Fig. 5j). In contrast, Wesseling et al. reported that ptau181 is present in AD and control (Braak stage < III) cases, as well as in seeding-competent and non-seeding-competent brain fractions [46]. While we observed a strong correlation of ptau181 with ptau212/214 across all hippocampus samples (Additional file 1: Fig. S4), ptau181 did not significantly correlate with tau seeding in Braak I-II cases (Fig. 5j, blue dots, Spearman's r_s_ = -0.61, p = 0.17). Even though these observations require verification in a larger sample set, they suggest that the levels of ptau212/214 could be a good indicator of early tau seeding activity in the brain.

We used two different techniques for the assessment of aggregated forms of tau: extraction and quantification of sarkosyl-insoluble tau and measurement of aggregated tau by symmetrical ELISA. Sarkosyl extraction is an established technique for the separation of large insoluble tau aggregates via multiple centrifugation steps [36]. Symmetrical ELISA can detect a range of smaller tau oligomers to larger tau aggregates by using the same HT7 antibody for capturing and detection [14]. Interestingly, the symmetrical ELISA detected aggregated tau in the majority of seeding-positive middle frontal gyrus samples from Braak II-III cases (Fig. 6b, g). In contrast, most of those brain samples had no detectable levels of sarkosyl-insoluble tau (Fig. 6c, h). These findings are consistent with the hypothesis that small aggregates of tau act as the initial seed ultimately leading to larger insoluble tau aggregates that manifest as inclusions at later Braak stages.

### Outlook towards tau SAA for accessible AD samples

The ability of our tau SAA to detect traces of tau seeds in AD brain render it potentially useful for applications where established cellular seeding assays for brain [12–15] face their limits, such as the translation into biofluids from AD patients [21]. Our tau SAA detected recombinant tau fibrils in the sub-pg/ml range (Fig. 2) and tau seeding in highly diluted brain samples (Fig. 3) approaching low fg/ml concentrations of ptau. The concentration of ptau in CSF is in the low pg/ml range [59] and therefore, in principle within the detectable range of our tau SAA. However, the extent to which ptau levels are related to the abundance of seeding-competent tau in CFS remains to be determined. In addition, testing of low dilutions of CSF or other matrices could lead to reduction of the SAA signal. Thus, optimization of tau SAA for the detection of seeding in accessible AD samples will be required and that is something we are currently exploring. The results obtained indicate that our development and in-depth characterization of a sensitive and specific tau SAA for AD brain provides an important step towards the analysis of tau seeding in accessible samples from AD patients.

### Supplementary Information

**Additional file 1. Figure S1.** Consistent tau seeding activity between two tau substrate batches. **Figure S2.** Individual ThT kinetic curves for all samples from cohort 1 sorted by brain area. **Figure S3.** Stratification of biochemical data for hippocampus samples based on tau seeding. **Figure S4.** Spearman correlation of ptau212/214 and ptau181 levels in hippocampus samples showed similar results. **Figure S5.** Tau parameters at individual Braak stages. **Figure S6.** Spearman correlation of 0N3R-tau-SAA with aggregated tau, sarkosyl-insoluble tau, ptau212/214 and total tau levels for cohort 3. **Table S1.** Summary of tau SAA substrates tested with Alzheimer’s disease (AD) and control (CTR) brain homogenate. **Table S2.** Detailed overview of neuropathology and donor demographics for cohort 1. **Table S3.** Detailed overview of neuropathology and donor demographics for cohort 2. **Table S4.** Detailed overview of neuropathology and donor demographics for cohort 3.
