# Correction: Tau seed amplification assay reveals relationship between seeding and pathological forms of tau in Alzheimer’s disease brain

**DOI:** 10.1186/s40478-024-01825-9

**Published:** 2024-07-15

**Authors:** Bryan Frey, David Holzinger, Keenan Taylor, Dagmar E. Ehrnhoefer, Andreas Striebinger, Sandra Biesinger, Laura Gasparini, Michael J. O’Neill, Florian Wegner, Stefan Barghorn, Günter U. Höglinger, Roland G. Heym

**Affiliations:** 1 AbbVie Deutschland GmbH & Co. KG, Neuroscience Research, Knollstrasse, 67061 Ludwigshafen, Germany; 2 AbbVie Bioresearch Center, Biotherapeutics and Genetic Medicine Technologies, Worcester, MA USA; 3 Department of Neurology, Hannover Medical School, Hanover, Germany; 4 Center for Systems Neuroscience, Hannover, Germany; 5 German Center for Neurodegenerative Diseases E.V. (DZNE), Munich, Germany; 6 Department of Neurology, LMU University Hospital, Ludwig-Maximilians-University (LMU), Munich, Germany

**Correction: Acta Neuropathologica Communications (2023) 11:181**

10.1186/s40478-023-01676-w

Following publication of the original article [1], the author noticed the errors in Table S1 of the Supplementary file.

In Table S1, the text part ‘C-terminal His6-tag’ should have read as ‘N-terminal His6-tag’ for Senostic Health GmbH.

The original article has been corrected.

### Electronic supplementary material

Below is the link to the electronic supplementary material.

Supplementary Material 1
