# Supplementary material for: Tau seed amplification assay reveals relationship between seeding and pathological forms of tau in Alzheimer’s disease brain
Source: Acta Neuropathol Commun. 2023 Nov 14;11:181. doi: 10.1186/s40478-023-01676-w (PMC10644662; doi:10.1186/s40478-023-01676-w)
Supplement: Supplementary file 1 — Additional file 1. Figure S1. Consistent tau seeding activity between two tau substrate batches. Figure S2. Individual ThT kinetic curves for all samples from cohort 1 sorted by brain area. Figure S3. Stratification of biochemical data for hippocampus samples based on tau seeding. Figure S4. Spearman correlation of ptau212/214 and ptau181 levels in hippocampus samples showed similar results. Figure S5. Tau parameters at individual Braak stages. Figure S6. Spearman correlation of 0N3R-tau-SAA with aggregated tau, sarkosyl-insoluble tau, ptau212/214 and total tau levels for cohort 3. Table S1. Summary of tau SAA substrates tested with Alzheimer’s disease (AD) and control (CTR) brain homogenate. Table S2. Detailed overview of neuropathology and donor demographics for cohort 1. Table S3. Detailed overview of neuropathology and donor demographics for cohort 2. Table S4. Detailed overview of neuropathology and donor demographics for cohort 3. [file 40478_2023_1676_MOESM1_ESM.docx]

**FIGURES**


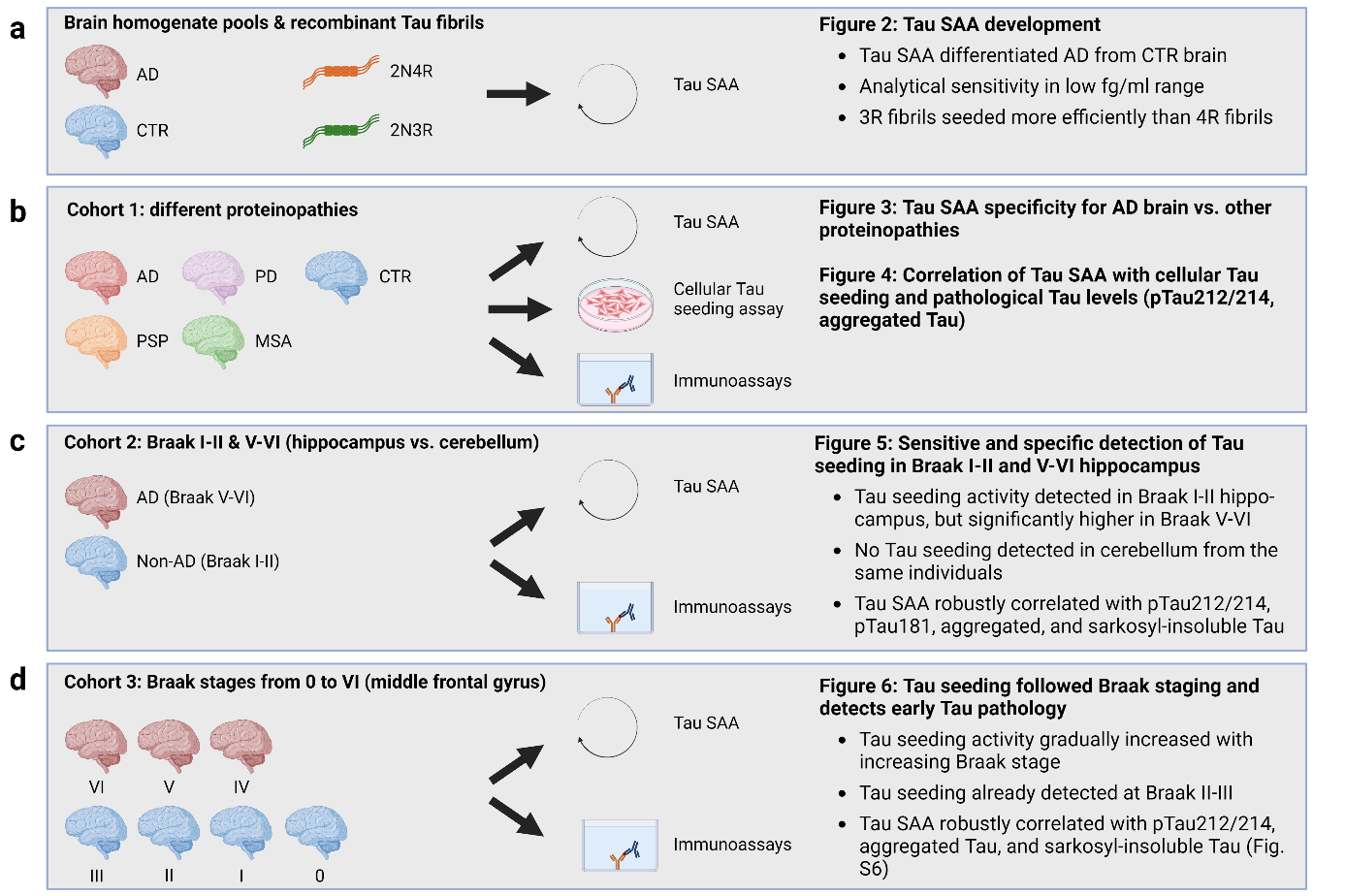


**Figure 1: Overview of study design and main results**. **a.** Figure 2. **b.** Figure 3 and 4. **c.** Figure 5. **d.** Figure 6. Roman numbers (I-VI) refer to the neuropathologically defined Braak stage. 2N4R and 2N3R refer to the Tau isoform used as recombinant Tau fibrils (Fig. 2). Tau SAA: Tau seed amplification assay, AD: Alzheimer’s disease, CTR: controls, PSP: Progressive Supranuclear Palsy, PD: Parkinson’s disease, MSA: Multiple System Atrophy.

| 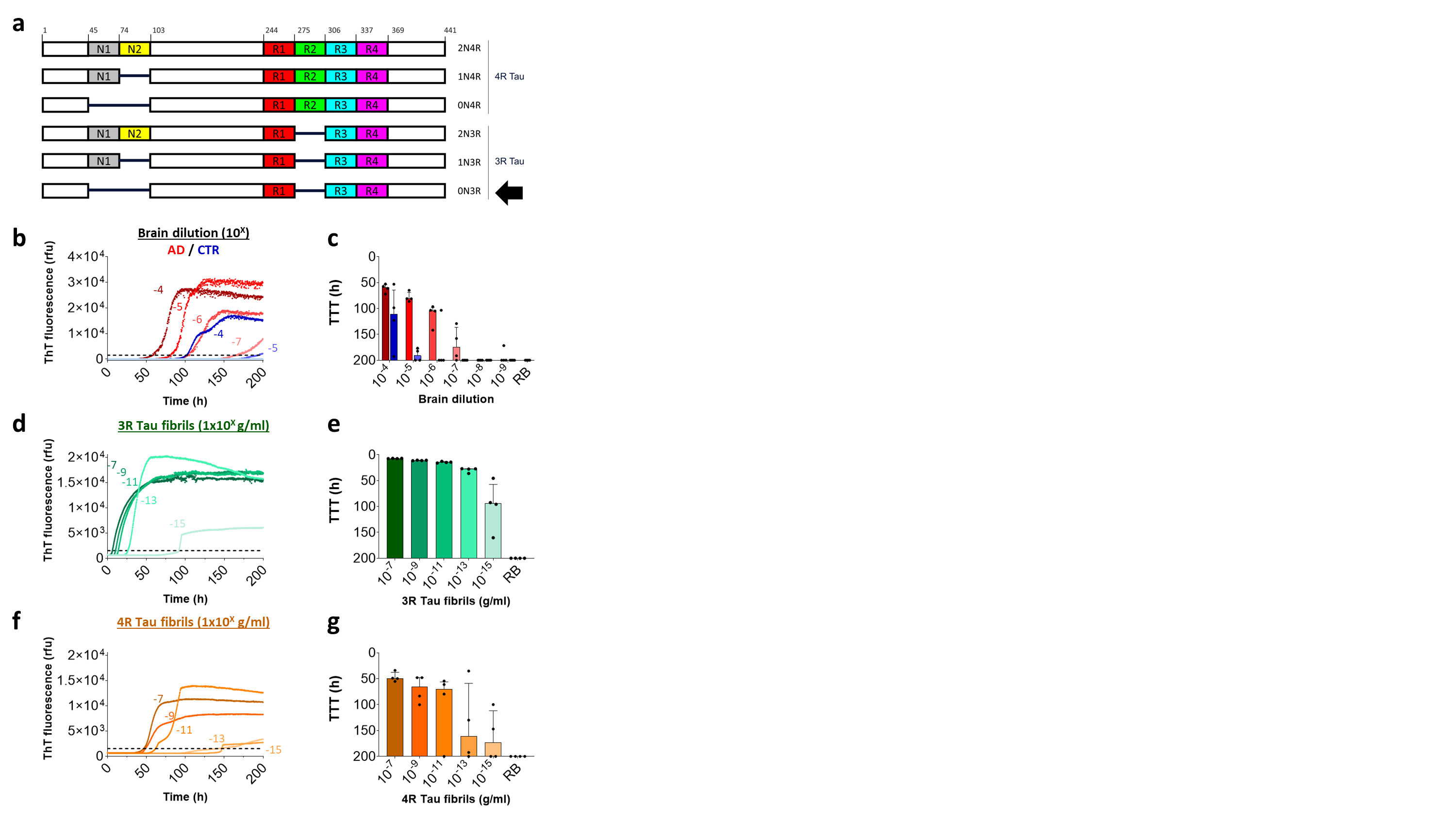 |
| --- |
| **Figure 2:** **Tau SAA detected seeding by AD brain homogenate and recombinant Tau fibrils with very high sensitivity.** **a.** Illustration of the six Tau isoforms in the human brain with amino-terminal domains N1 and N2 and repeat domains R1-R4. Amino acid numbering on the top is referring to the 2N4R isoform. Arrow indicates the 0N3R Tau isoform used in Tau SAA. **b.** Thioflavin T (ThT) kinetic curves of serially diluted AD (red) vs. control (CTR, blue) brain pools. Dotted lines depict the threshold of 1500 relative fluorescent units (rfu) for determination of the time to threshold (TTT) value. Curves are medians of technical quadruplicates. Numbering indicates dilution factor. **c.** TTT values derived from curves shown in b. Each dot depicts a technical replicate, bars are medians, and error bars show the interquartile range. RB: reaction buffer. **d-e.** Tau SAA results of 2N3R Tau (green) fibrils. **f-g.** Tau SAA results of 2N4R (orange) Tau fibrils. |
| 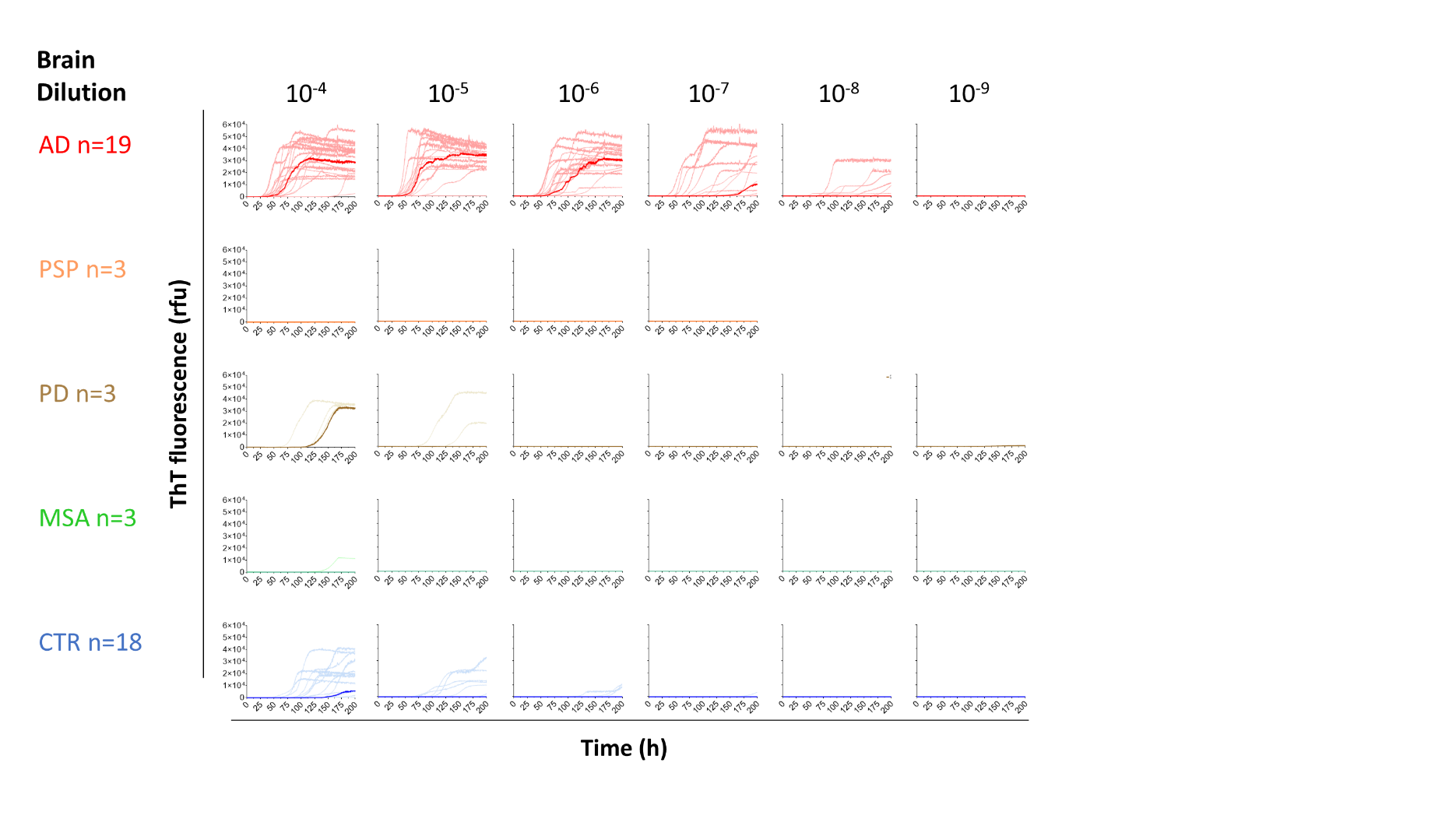  **Fig. 3:** **Tau SAA showed high specificity for AD compared to Progressive Supranuclear Palsy (PSP), Parkinson’s disease (PD), Multiple System Atrophy (MSA) and control (CTR ) brains from cohort 1.** Graphs show ThT kinetic curves of serially diluted AD (red, n=19), PSP (orange, n=3), PD (brown, n=3), MSA (green, n=3) and control (cyan, n= 18) brain homogenates. Dark colored curves depict the group median and light colored curves show the median of 3-4 technical replicates for each individual sample. Cohort 1 is described in detail in Table 1 and S2. |

| 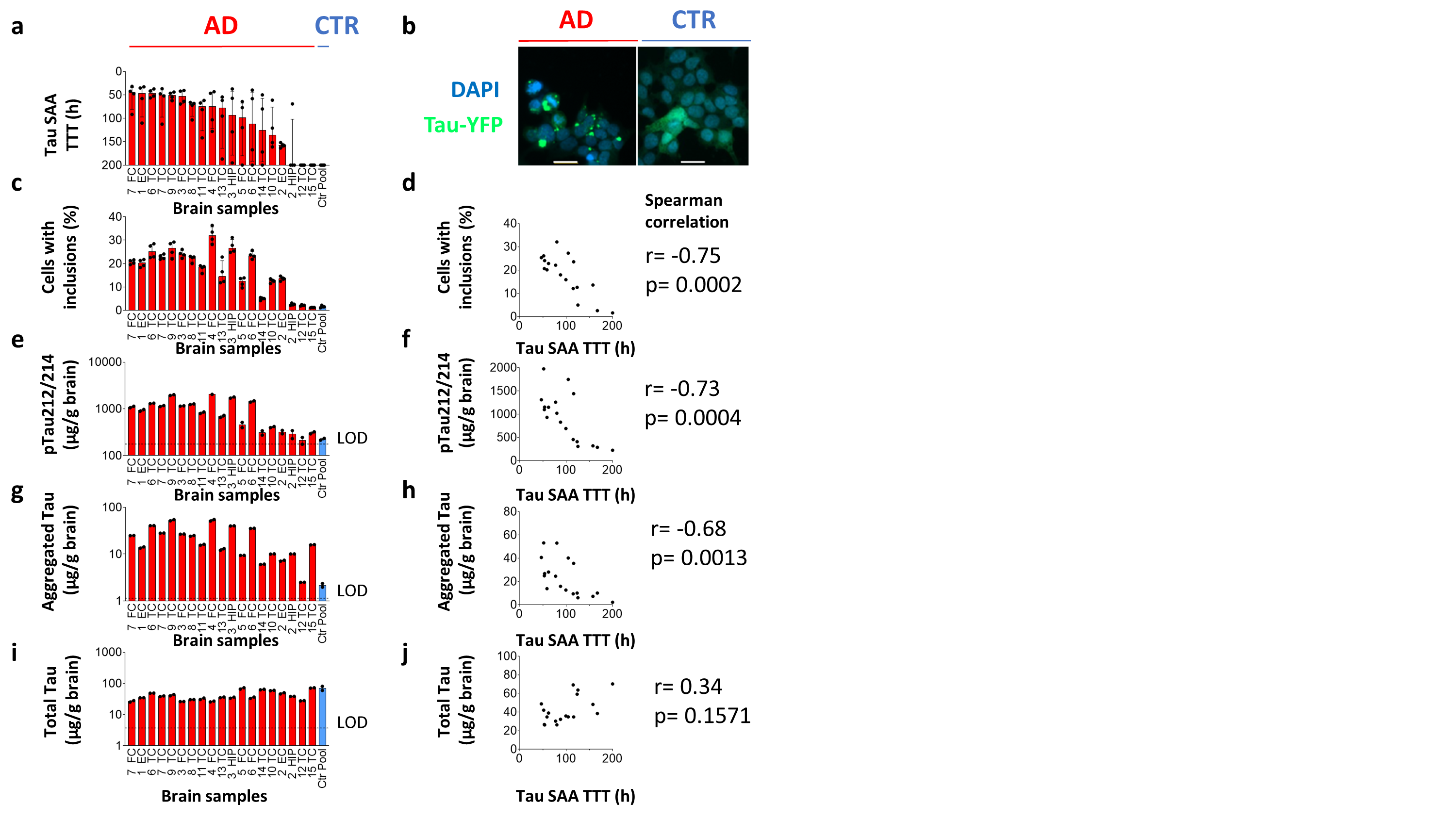 |
| --- |
| **Fig. 4:** **Tau SAA TTT of AD brain homogenates from cohort 1 correlated with Tau biosensor cell seeding assay, phosphorylated Tau, and aggregated Tau.** **a.** Nineteen AD brains (red) and one control brain pool (CTR, blue) were analyzed with Tau SAA. Tau SAA was performed with brain homogenates diluted 10^-6^. X-axis indicates case numbers and brain regions (EC: Entorhinal cortex; FC: Frontal Cortex; HIP: Hippocampus; TC: Temporal Cortex). Dots represent technical replicates. Bars and error bars indicate medians and interquartile ranges. **b.** Representative images from Tau biosensor cell seeding assay. HEK293 cells stably expressing Tau repeat domains (amino acids 244-372) with P301S mutation fused to yellow fluorescent protein (Tau-YFP) were incubated with AD and CTR brain pools for 48h. Afterwards, cells were fixed, stained with DAPI and imaged for Tau-YFP (green) and DAPI (blue). Scale bar represents 15 µm. **c.** Quantification of YFP-positive inclusions in Tau biosensor cell seeding assay incubated with brain homogenates diluted 1:8. **d.** Spearman correlation of Tau SAA TTT with percent of cells with inclusions in Tau biosensor cell seeding assay.  **e-j.** Quantification of pTau212/214, aggregated Tau and total Tau by ELISA, and their Spearman correlation with Tau SAA TTT. Dotted lines show limits of detection (LOD). Cohort 1 is described in detail in Table 1 and S2. |
| 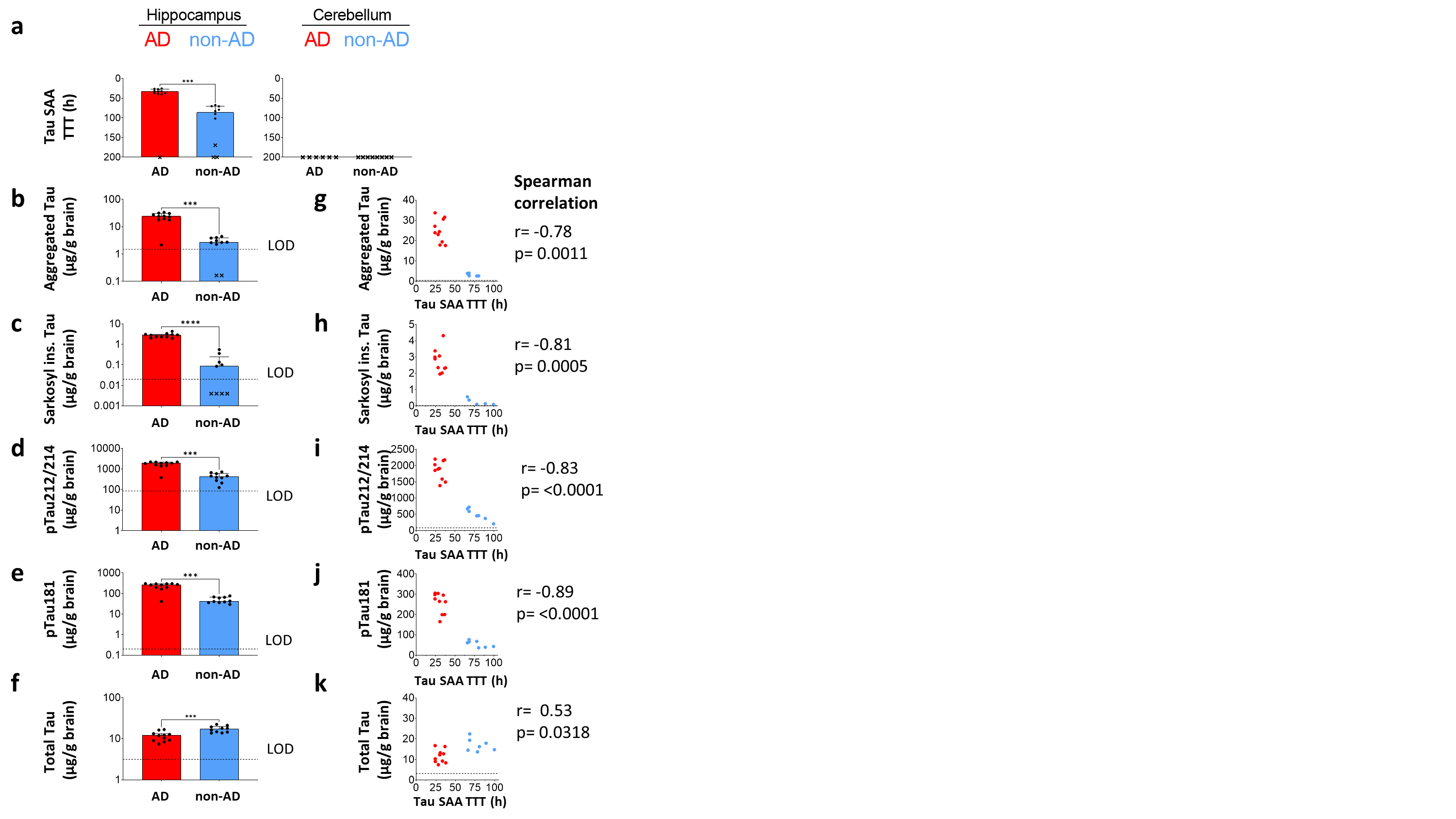 |
| **Fig. 5: Sensitive detection of pathological and seeding-competent Tau forms in AD (Braak V-VI) and non-AD (Braak I-II) hippocampus. a.** Tau-SAA time to threshold (TTT) values of AD (Braak V-VI) and non-AD (Braak I-II) hippocampus (Hip) and cerebellum (Cer) homogenates (see Table S3), diluted by 10^-6^. Dots indicate medians of technical quadruplicates per brain; crosses: non-seeding samples with at least 3 out of 4 technical replicates showing TTT values ≥200 h. **b-f.** Immunoassay results for aggregated Tau, sarkosyl-insoluble Tau, pTau212/214, pTau181, and total Tau. Dots: mean of technical duplicates. Dotted lines indicate the limit of detection (LOD). Bars: group medians + interquartile range. Statistical comparisons of group medians were performed by Mann-Whitney test. *p<0.05, **p <0.01, ***p<0.001. **g-k.** Spearman correlation of Tau-SAA TTT versus immunoassay results. Non-seeding samples and values below the LOD were excluded. Cohort 2 is described in detail in Table 2 and S3. |


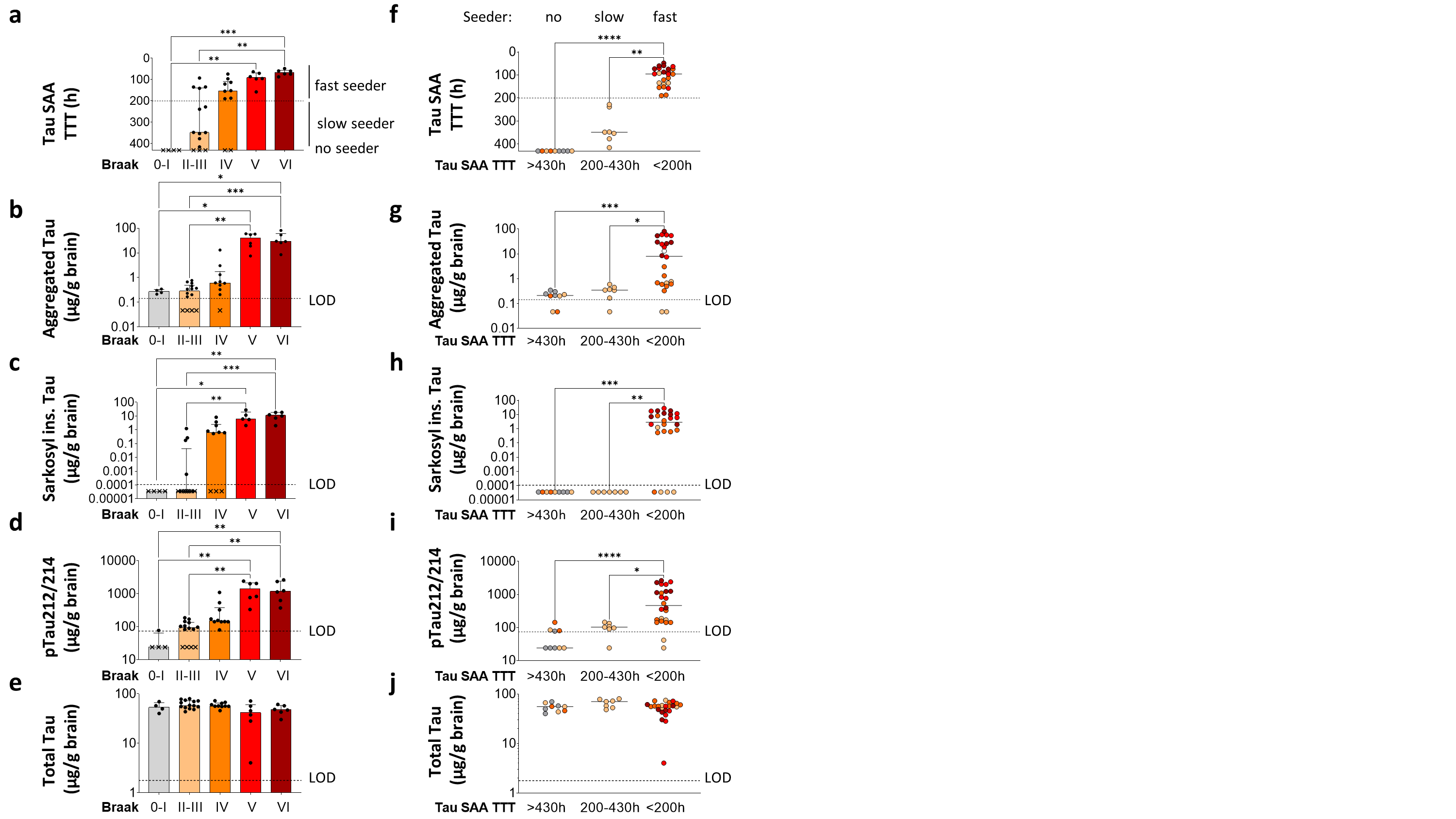


**Fig. 6: Tau-SAA seeding activity in middle frontal gyrus gradually increased with Braak stage (0-VI) and reflected the load of pathological Tau forms.** **a.** Time to threshold (TTT) values from Tau SAA of middle frontal gyrus homogenates (see Table S4) diluted 10^-5^. Dotted line: separating fast-seeding (TTT < 200 h) and slow-seeding samples (TTT of 200-430 h). Crosses: non-seeding samples (TTT of ≥430h). Dots depict the mean TTT values of technical triplicates. Bars represent the group medians + interquartile range. Statistical comparisons by Kruskal-Wallis test with Dunn’s multiple comparisons correction. *p<0.05, **p <0.01, ***p< 0.001. **b-e.** ELISA results of aggregated Tau, sarkosyl-insoluble Tau, pTau212/214, and total Tau. Dots depict the mean of technical duplicates. Crosses in indicate values below the limit of detection (LOD, dotted line) and were set to one third of the LOD. **f-j** Stratification of samples into fast- (TTT <200 h), slow- (TTT of 200 h–430 h) and no seeding (TTT of ≥430h) based on Tau SAA. Braak stages are colored as indicated in a-e. Vertical lines represent the group medians. Statistical comparisons by Kruskal-Wallis test with Dunn’s multiple comparisons correction. *p<0.05, **p <0.01, ***p< 0.001. Cohort 3 is described in detail in Table 3 and S4.

**SUPPLEMENTARY FIGURES**


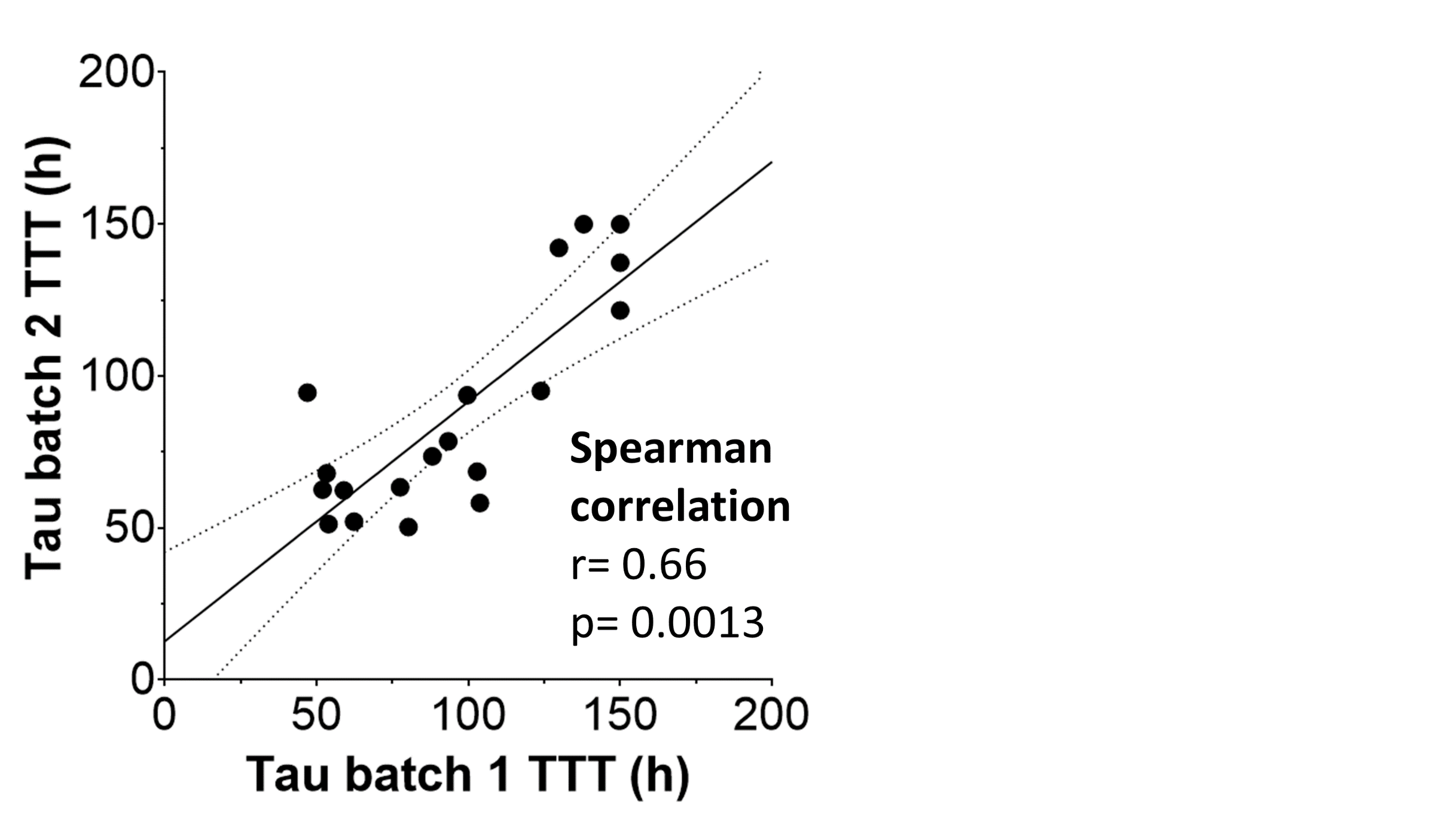


**Figure S1:** **Consistent Tau seeding activity between two Tau substrate batches.** Spearman correlation of two Tau SAA runs with different purification batches of 0N3R-Tau (C322S) substrate. Dots represent median TTT values (Batch 1: technical quadruplicate, Batch 2: technical triplicates) of AD brains listed in Table S2. Line depicts a linear fit of the data points.

| 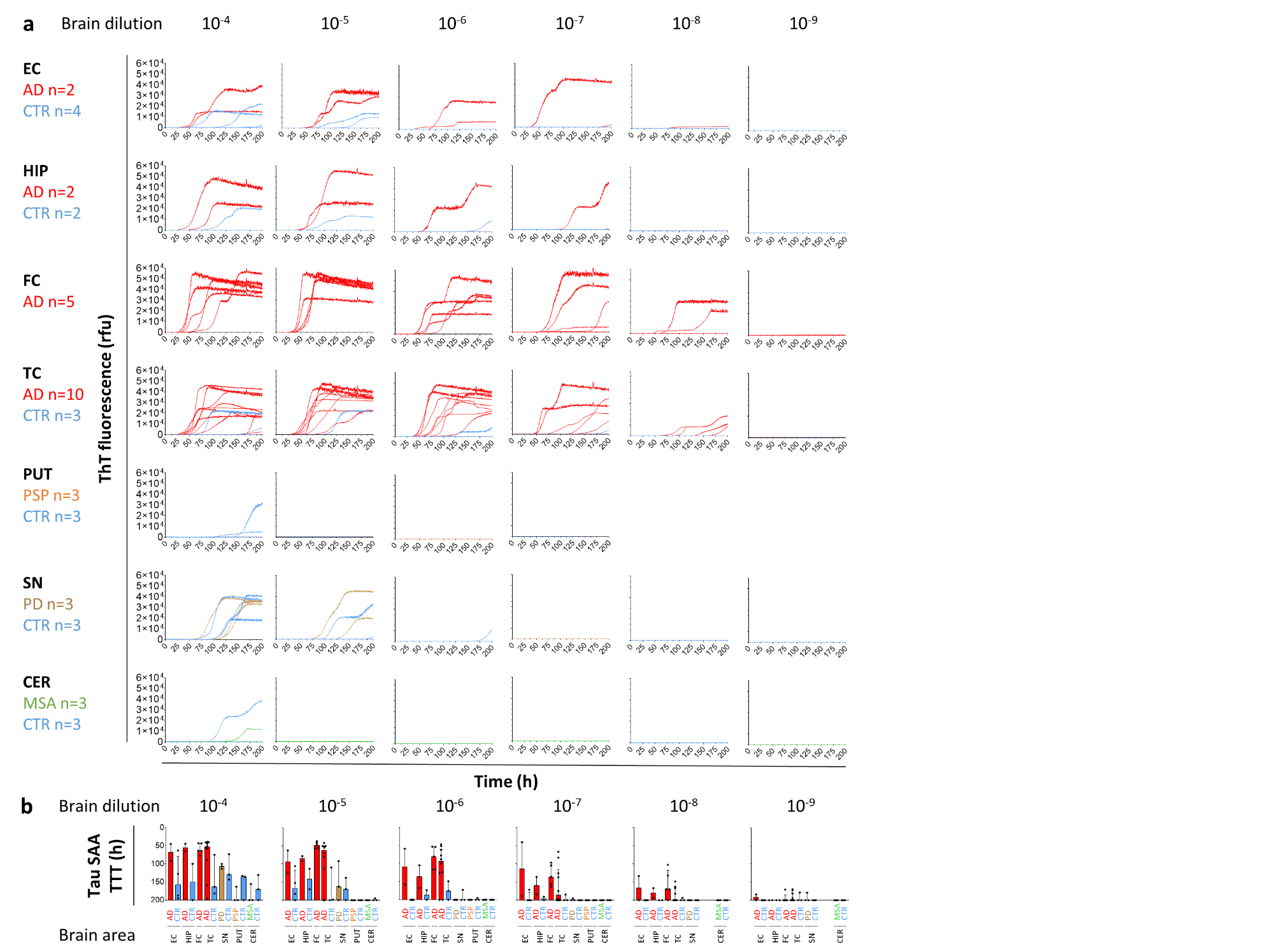 |
| --- |
| **Figure S2: Individual ThT kinetic curves for all samples from cohort 1 sorted by brain area.**  **a.** ThT kinetic curves represent the median of 3-4 technical replicates per sample dilution. Abbreviations: AD: Alzheimer’s disease (red), CTR: Control (blue), PD: Parkinson’s disease (brown), PSP: Progressive Supranuclear Palsy (orange), MSA: Multiple System Atrophy (green); EC: Entorhinal cortex, HIP: Hippocampus, FC: Frontal cortex, TC: Temporal cortex, PUT: Putamen, SN: Substantia nigra, CER: Cerebellum. **b.** TTT values derived from ThT kinetic curves shown in a. Dots represent median TTT from 3-4 technical replicates per sample. Bars and error bars show the group median and interquartile range. Cohort 1 is described in detail in Table 1 and S2. |


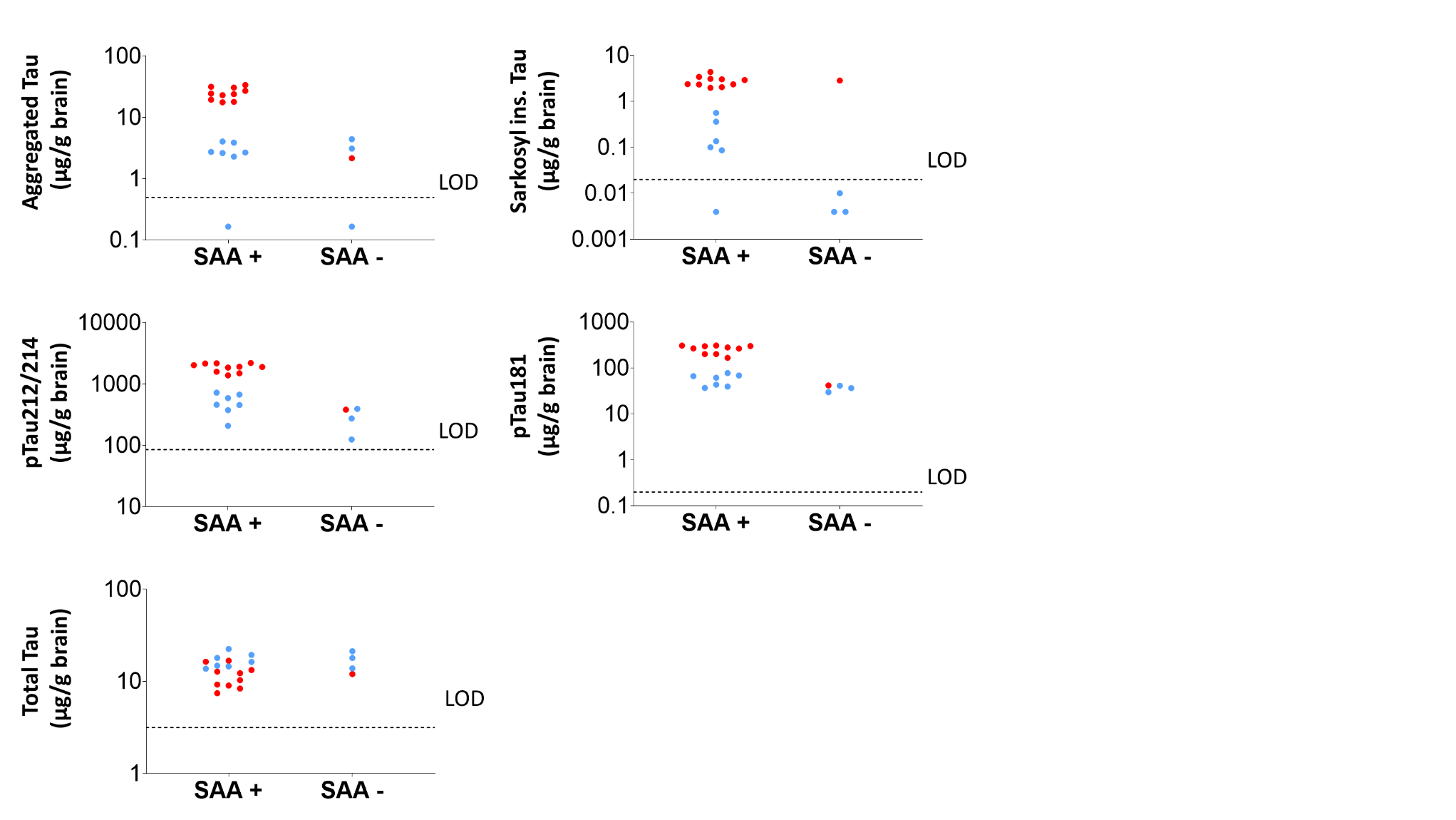


**Fig S3: Stratification of biochemical data for hippocampus samples based on Tau seeding.** Hippocampus samples were separated into seeding (<200 h) and non-seeding samples (≥200 h) based on Tau SAA: TTT values and ELISAs concentrations are shown. Each data point represents the median of technical quadruplicates (Tau SAA) or mean of technical duplicates (ELISA). Dotted line represents the limit of detection of the respective assay. Values below the detection limit were set to one third of the limit of detection. Red: Alzheimer’s disease. Blue: non-AD. Cohort 2 is described in detail in Table 2 and S3.


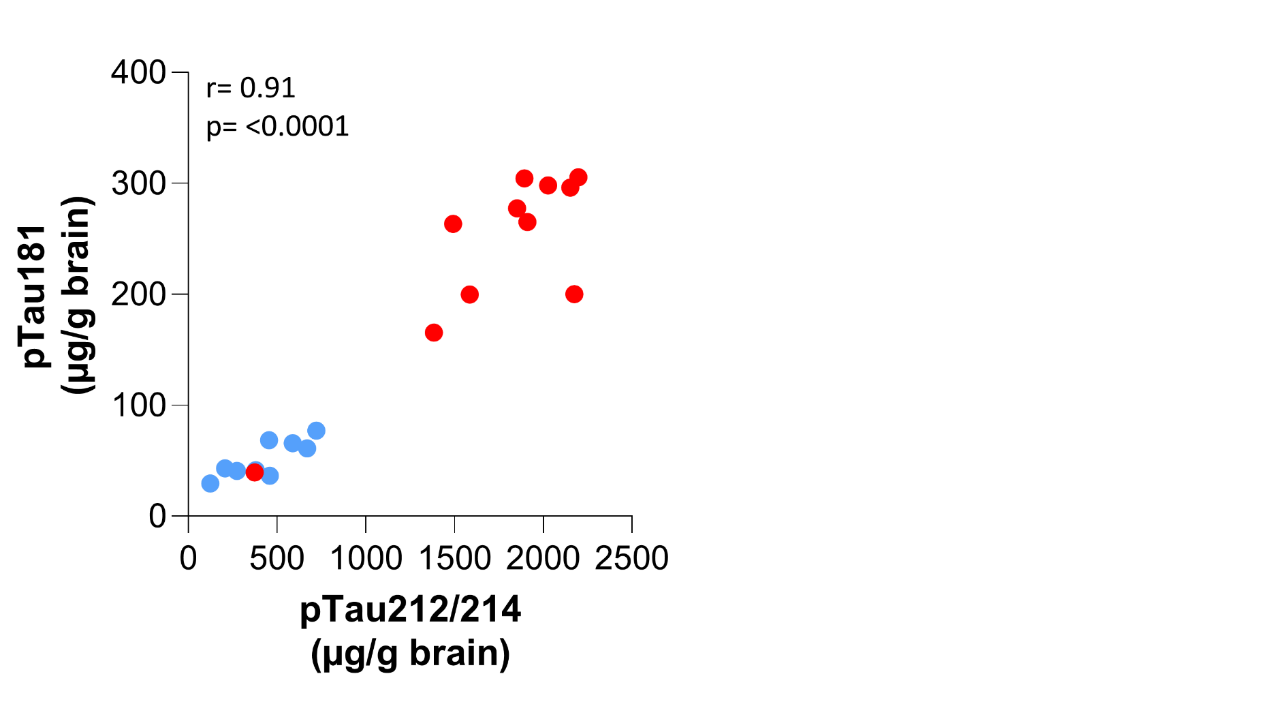


**Fig. S4: Spearman correlation of pTau212/214 and pTau181 levels in hippocampus samples showed similar results.** Blue dots: non-AD cases, Red: Alzheimer’s disease. Cohort 2 is described in detail in Table 2 and S3.


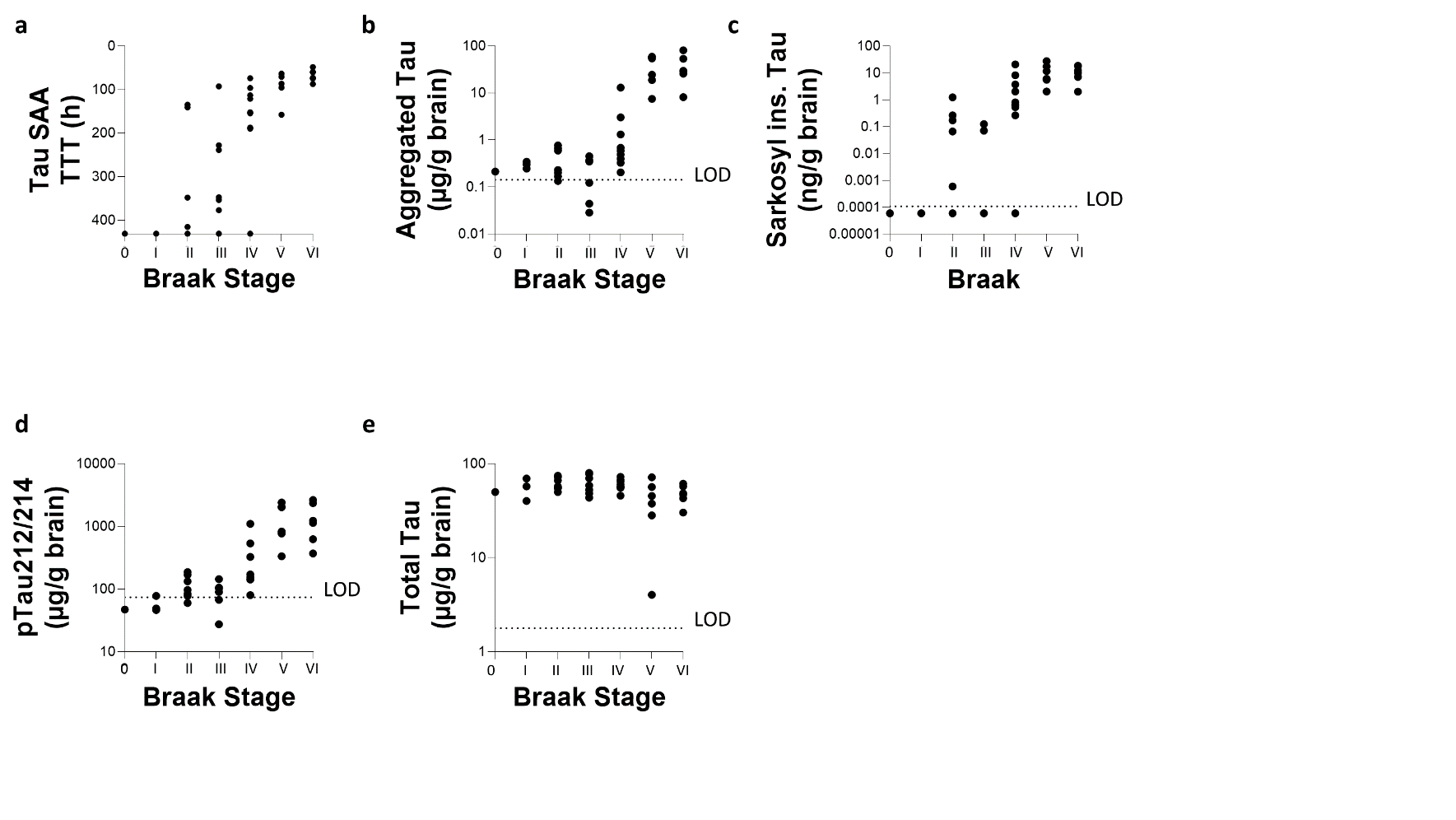


**Fig S5: Tau parameters at individual Braak stages.** Braak stage vs **a.** Tau SAA Time to Threshold (TTT); **b.** aggregated Tau; **c.** sarkosyl insoluble Tau; **d.** pTau212/214 and **e.** total Tau. Dots: individual brain samples. Cohort 3 is described in detail in Table 3 and S4. Dotted line represents the limit of detection of the respective assay.


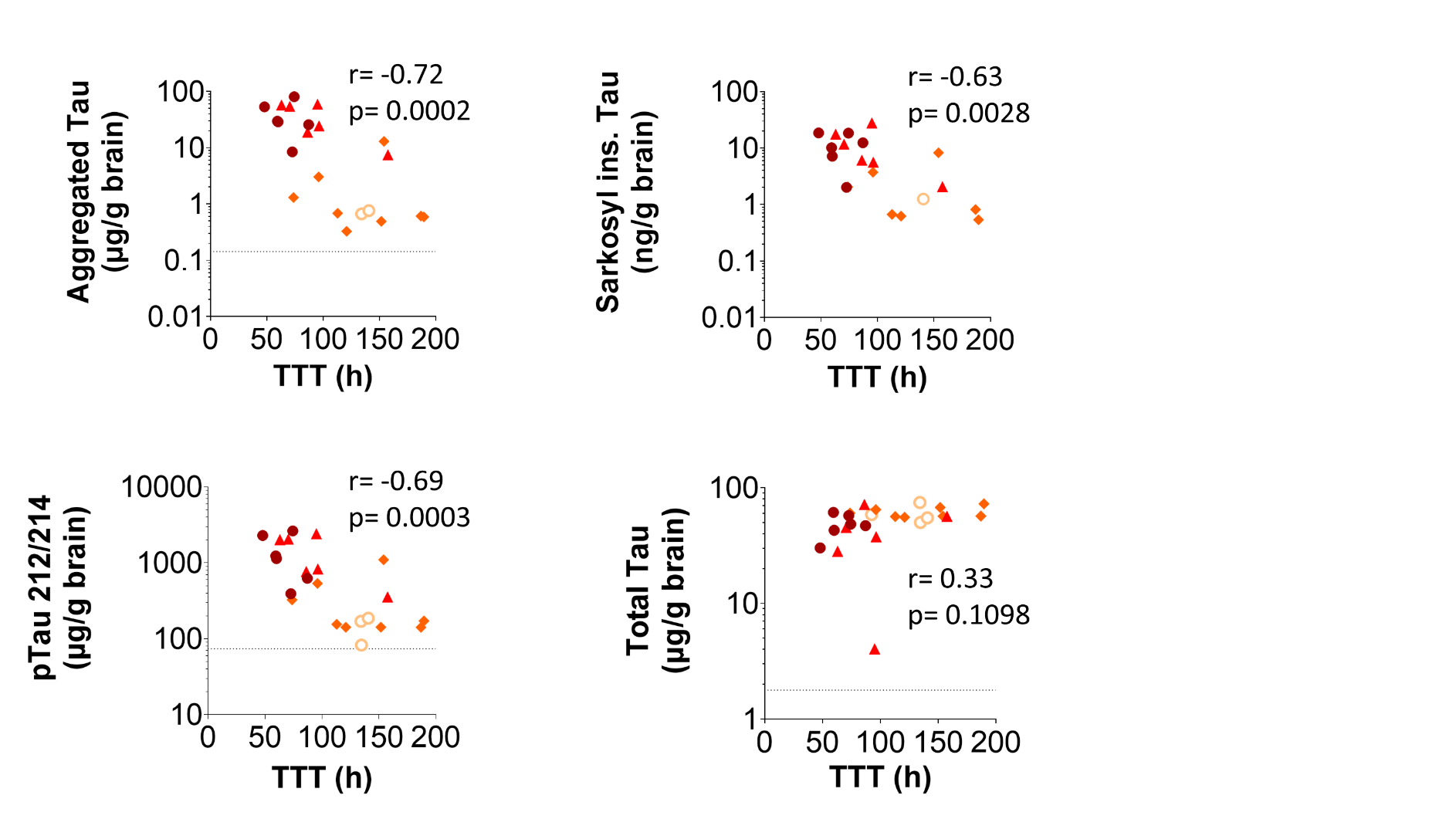


**Fig S6: Spearman correlation of 0N3R-Tau-SAA with aggregated Tau, sarkosyl-insoluble Tau, pTau212/214 and total Tau levels for cohort 3.** Correlation analysis of data from Fig. 5. To be consistent with analyses of cohorts 1 and 2, we restricted the analysis to fast-seeding samples with TTT < 200 h. Dark Red dots (●): Braak stage VI, light red triangles (▲): Braak stage V, Dark orange diamonds (⯁): Braak stage IV, light orange circles (○): Braak stage II-III. Cohort 3 is described in detail in Table 3 and S4.

**TABLES**

| **Table 1:** **Basic characteristics of cohort 1.** Alzheimer’s disease (AD), Progressive supranuclear palsy (PSP), Parkinson’s disease (PD), Multiple system atrophy (MSA) and control (CTR) brain samples. Four AD patients donated two different brain areas (for details see Table S1). Old, non-demented controls had neurofibrillary tangles classifying them as Braak I-II. EC: Entorhinal cortex; HIP: Hippocampus; FC: Frontal Cortex; TC: Temporal Cortex; PUT: Putamen; SN: Substantia Nigra; CER: Cerebellum; y: years; f: female; m: male; n.d.: not determined. *Indicated Braak stage (Tau) based on 3 C donors. For detailed description of cohort 1 samples, see Table S2 |
| --- |

| **Disease** | **N** | **Age, mean and range (y)** | **Braak (Tau)** | **Sex  (f/m)** | **Region** | **N** | **Figure** |
| --- | --- | --- | --- | --- | --- | --- | --- |
| AD | 15 | 69 (55 – 86) | V-VI | 11/4 | EC | 2 | 3, 4 |
|  |  |  |  |  | HIP | 2 |  |
|  |  |  |  |  | FC | 5 |  |
|  |  |  |  |  | TC | 10 |  |
| PSP | 3 | 80 (72-89) | n.d. | 1/2 | PUT | 3 |  |
| PD | 3 | 80 (74-90) | 0-II | 0/3 | SN | 3 |  |
| MSA | 3 | 61 (60-62) | n.d. | 3/0 | CER | 3 |  |
| CTR | 19 | 74 (49-93) | I-II* | 8/11 | EC | 4 |  |
|  |  |  |  |  | HIP | 3 |  |
|  |  |  |  |  | TC | 3 |  |
|  |  |  |  |  | PUT | 3 |  |
|  |  |  |  |  | SN | 3 |  |
|  |  |  |  |  | CER | 3 |  |

| **Table 2:** **Basic characteristics of cohort 2.** Hippocampus and cerebellum of AD (Braak V-VI) and non-AD (Braak I-II) donors including 6 AD and 8 non-AD cases with matched samples from the same donors. HIP: Hippocampus; CER: Cerebellum; AD: Alzheimer’s disease; y: years; f: female; m: male. For detailed description of cohort 2 samples, see Table S3 |
| --- |

| **Disease** | **Region** | **N** | **Age mean and range (y)** | **Braak (Tau)** | **Sex**  **(f/m)** | **Figure** |
| --- | --- | --- | --- | --- | --- | --- |
| AD | HIP | 11 | 72 (58-84) | V-VI | 6/5 | 5 |
| AD | CER | 6 | 72 (58-84) | V-VI | 3/3 |  |
| Non-AD | HIP | 10 | 86 (70-95) | I-II | 6/4 |  |
| Non-AD | CER | 8 | 87 (75-95) | I-II | 5/3 |  |

| **Table 3:** **Basic characteristics of cohort 3.** Middle frontal gyrus from 40 brains ranging from Braak 0 to VI. y: years; f: female; m: male. For detailed description of cohort 4 samples, see Table S3 |
| --- |

| **Cases (Braak)** | **N** | **Sex**  **(f/m)** | **Age, mean and range (y)** | **Figure** |
| --- | --- | --- | --- | --- |
| 0 - I | 4 | 3/1 | 86 (78-93) | 6 |
| II - III | 14 | 11/3 | 87 (76-99) |  |
| IV | 10 | 7/3 | 88 (72-100) |  |
| V | 6 | 3/3 | 75 (70-82) |  |
| VI | 6 | 4/2 | 70 (55-84) |  |

**SUPPLEMENTARY TABLES**

**Table S1: Summary of Tau SAA substrates tested with Alzheimer’s disease (AD) and control (CTR) brain homogenate.** TTT: time to threshold value from Tau SAA based brains diluted by 10^-5^.

| **Category** | **Source** | **Tau isoform** | **Mutations** | **Tag** | **Self-aggregation** "-" = no aggregation "+" = aggregation | **Tau SAA results: ratio TTT (CTR) / TTT (AD))** "-" = no difference "+" = 1.1-1.5-fold difference  "+++" = >2.5-fold difference |
| --- | --- | --- | --- | --- | --- | --- |
| Full-length 4R | In-house | 2N4R |  | untagged | - | - |
|  | In-house | 2N4R | C291A, C322A | untagged | - | - |
|  | In-house | 2N4R | ∆K280, C291A, C322A | untagged | - | - |
|  | In-house | 0N4R |  | untagged | - | - |
|  | Senostic Health GmbH | 0N4R | C291S, C322S | N-terminal His6-tag | + | + |
| Full-length 3R | In-house | 2N3R | C322A | untagged | - | - |
|  | In-house | 0N3R |  | untagged | - | - |
|  | In-house | 0N3R | C322S | untagged | - | - |
|  | Senostic Health GmbH | 0N3R | C322S | N-terminal His6-tag | - | +++ |
| Full-length 4R/3R ratio 1:1 | In-house | 2N4R 2N3R | C291A, C322A C322A | untagged | - | - |
|  | Senostic Health GmbH | 0N4R 0N3R | C291S, C322S C322S | N-terminal His6-tag N-terminal His6-tag | + | + |

| **Table S2:** **Detailed overview of neuropathology and donor demographics for cohort 1.**  Tau substrate batch 1 was used (see SFig 1). *: Results generated by separate measurement of two tissue punches from the same brain area of one donor using the median TTT; Tau SAA TTT: Median time to threshold of quadruplicate measures from brain dilution of 10^-6^. COD: Cause of death; Braak Stage (NFTs): Braak staging for Tau neurofibrillary tangles; Braak Stage (LB): Braak staging for Lewy bodies; PMD: Post-mortem delay; AD: Alzheimer’s disease; PSP: Progressive supranuclear palsy; PD: Parkinson’s disease; MSA: Multiple system atrophy; CTR: Controls; Dementia-NOS: not otherwise specified; EC: Entorhinal cortex; HIP: Hippocampus; FC: Frontal Cortex; TC: Temporal Cortex; PUT: Putamen; SN: Substantia Nigra; CER: Cerebellum; y: years; m: male; f: female; n.a.: not available. ABS: Analytical Biological Services Inc.; Banner: Banner sun health research institute; TS: Tissue Solutions Ltd.; NBB: Netherlands brain bank; DLS: Discovery life sciences; FB: Folio Biosciences.   \| **Case**  **#** \| **Neuropathological diagnosis** \| **Clinical Diagnosis** \| **COD** \| **Braak**  **(NFTs)** \| **Braak**  **(Lewy Bodies)** \| **Sex** \| **Age**  **(y)** \| **PMD**  **(h)** \| **Source** \| **Brain**  **Region** \| **Tau SAA**  **TTT (h)** \| **Positive replicates**  **(%)** \| \| --- \| --- \| --- \| --- \| --- \| --- \| --- \| --- \| --- \| --- \| --- \| --- \| --- \| \| 1 \| AD \| AD \| Respiratory failure, Pneumonia \| VI \| n.a. \| m \| 71 \| 4.6 \| TS \| EC \| 59 \| 100 \| \| 2* \| AD \| AD \| Dementia \| V-VI \| n.a. \| f \| 86 \| 4 \| ABS \| EC \| 158 \| 100 \| \| HIP \| 200 \| 25 \| \| 3 \| AD \| Advanced AD \| Cardiopulmonary arrest \| V-VI \| n.a. \| f \| 59 \| 18.5 \| TS \| HIP \| 105 \| 100 \| \| FC \| 54 \| 100 \| \| 4 \| AD \| AD with behavioral disturbance \| Congestive Heart Disease, Coronary Artery Disease,  Cerebral Degeneration \| V-VI \| n.a. \| f \| 60 \| 23 \| TS \| FC \| 80 \| 100 \| \| 5 \| AD \| AD \| Presumed AD \| V-VI \| n.a. \| f \| 86 \| 11.2 \| TS \| FC \| 128 \| 75 \| \| 6 \| AD \| AD,  Arteriolosclerosis \| Dementia \| V-VI \| n.a \| f \| 55 \| 19 \| TS \| FC \| 118 \| 175 \| \| TC \| 47 \| 100 \| \| 7 \| AD \| Dementia-other \| AD \| V-VI \| n.a. \| f \| 60 \| 20.5 \| TS \| FC \| 53 \| 100 \| \| TC \| 62 \| 100 \| \| 8* \| AD \| AD \| n.a. \| V-VI \| n.a. \| f \| 60 \| n.a. \| DLS \| TC \| 78 \| 100 \| \| 9 \| AD \| AD \| n.a. \| VI \| n.a. \| f \| 69 \| n.a. \| DLS \| TC \| 52 \| 100 \| \| 10 \| AD \| AD \| n.a. \| V-VI \| n.a. \| f \| 59 \| n.a. \| DLS \| TC \| 124 \| 100 \| \| 11* \| AD \| AD \| n.a. \| V-VI \| n.a. \| f \| 84 \| n.a. \| DLS \| TC \| 88 \| 100 \| \| 12* \| AD \| AD \| n.a. \| VI \| n.a. \| m \| 70 \| n.a. \| DLS \| TC \| 200 \| 0 \| \| 13* \| AD \| AD \| n.a. \| VI \| n.a. \| m \| 74 \| n.a. \| DLS \| TC \| 100 \| 100 \| \| 14 \| AD \| AD \| AD \| V-VI \| n.a. \| f \| 70 \| 3 \| ABS \| TC \| 138 \| 75 \| \| 15 \| AD \| AD \| AD \| V-VI \| n.a. \| m \| 79 \| 3 \| ABS \| TC \| 200 \| 0 \| \| 16 \| PSP \| PSP \| PSP \| n.a. \| n.a. \| m \| 78 \| 15 \| TS \| PUT \| 200 \| 0 \| \| 17 \| PSP \| PSP \| Parkinsonism syndrome, PSP \| n.a. \| n.a. \| m \| 89 \| 13.5 \| TS \| PUT \| 200 \| 0 \| \| 18 \| PSP \| PSP \| Mixed Dementia \| n.a. \| n.a. \| f \| 72 \| 12.4 \| TS \| PUT \| 200 \| 0 \| \| 19 \| PD \| PD Dementia \| Failure to thrive \| n.a. \| n.a. \| m \| 75 \| 6 \| TS \| SN \| 200 \| 0 \| \| 20 \| PD \| PD \| Aspiration pneumonia \| n.a. \| IV \| m \| 90 \| 21 \| TS \| SN \| 200 \| 0 \| \| 21 \| PD \| PD \| Complication of end stage PD \| n.a. \| n.a. \| m \| 74 \| 15 \| TS \| SN \| 200 \| 0 \| \| 22 \| MSA \| MSA, MCI \| n.a. \| III \| n.a. \| f \| 62 \| 4.6 \| Banner \| CER \| 200 \| 0 \| \| 23 \| MSA \| MSA \| n.a. \| II \| n.a. \| f \| 60 \| 3.9 \| Banner \| CER \| 200 \| 0 \| \| 24 \| MSA \| MSA, Dementia \| n.a. \| I \| n.a. \| f \| 62 \| 3.8 \| Banner \| CER \| 200 \| 0 \| \| 25 \| CTR \| Dementia NOS \| Hypertensive Heart Disease \| II \| n.a. \| m \| 75 \| 12 \| TS \| SN \| 200 \| 0 \| \| 26 \| CTR \| Unaffected \| Acute Myocardial infarction \| n.a. \| n.a. \| m \| 90 \| 15 \| TS \| SN \| 185 \| 75 \| \| 27 \| CTR \| Unaffected \| Abdominal Aortic Aneurysm \| n.a. \| n.a. \| m \| 74 \| 20 \| TS \| SN \| 200 \| 0 \| \| 28 \| CTR \| Unaffected \| Pending death certificate \| n.a. \| n.a. \| m \| 74 \| 23.3 \| TS \| PUT \| 200 \| 0 \| \| 29 \| CTR \| Unaffected \| Rupture of Abdominal Aortic Aneurysm \| n.a. \| n.a. \| m \| 74 \| 19.8 \| TS \| PUT \| 200 \| 0 \| \| 30 \| CTR \| Unaffected \| Malignant Arythmia \| n.a. \| n.a. \| m \| 73 \| 25.4 \| TS \| PUT \| 200 \| 0 \| \| 31 \| CTR \| Unaffected \| n.a. \| III \| n.a. \| m \| 71 \| 3.5 \| Banner \| CER \| 200 \| 0 \| \| 32 \| CTR \| Unaffected \| n.a. \| II \| n.a. \| m \| 79 \| 4.3 \| Banner \| CER \| 200 \| 0 \| \| 33 \| CTR \| Unaffected \| n.a. \| II \| n.a. \| f \| 82 \| 2 \| Banner \| CER \| 200 \| 33.3 \| \| 34 \| CTR \| Unaffected \| n.a. \| n.a. \| n.a. \| m \| 49 \| 10.5 \| FB \| TC \| 200 \| 25 \| \| 35 \| CTR \| Unaffected \| n.a. \| n.a. \| n.a. \| f \| 73 \| 4.7 \| FB \| TC \| 160 \| 75 \| \| 36 \| CTR \| Unaffected \| n.a. \| n.a. \| n.a. \| m \| 54 \| 14 \| FB \| TC \| 200 \| 0 \| \| 37 \| CTR \| Unaffected \| Cardiac Arrest \| n.a. \| n.a. \| m \| 83 \| 7.5 \| ABS \| EC \| 200 \| 0 \| \| 38 \| CTR \| Unaffected \| Pancreatic Cancer \| n.a. \| n.a. \| f \| 57 \| 3 \| ABS \| EC \| 200 \| 0 \| \| 39 \| CTR \| Unaffected \| Acute Myocardial infarction \| n.a. \| n.a. \| f \| 66 \| 2.5 \| ABS \| EC \| 200 \| 0 \| \| 40 \| CTR \| Unaffected \| Cancer \| n.a. \| n.a. \| f \| 78 \| 8 \| ABS \| EC \| 200 \| 0 \| \| 41 \| CTR \| Unaffected \| n.a. \| II \| 0 \| f \| 93 \| 7 \| NBB \| HIP \| 200 \| 0 \| \| 42 \| CTR \| Unaffected \| Cerebrovascular accident \| II \| n.a. \| f \| 85 \| 7 \| NBB \| HIP \| 189 \| 50 \| |
| --- | --- | --- | --- | --- | --- | --- | --- | --- | --- | --- | --- | --- | --- | --- | --- | --- | --- | --- | --- | --- | --- | --- | --- | --- | --- | --- | --- | --- | --- | --- | --- | --- | --- | --- | --- | --- | --- | --- | --- | --- | --- | --- | --- | --- | --- | --- | --- | --- | --- | --- | --- | --- | --- | --- | --- | --- | --- | --- | --- | --- | --- | --- | --- | --- | --- | --- | --- | --- | --- | --- | --- | --- | --- | --- | --- | --- | --- | --- | --- | --- | --- | --- | --- | --- | --- | --- | --- | --- | --- | --- | --- | --- | --- | --- | --- | --- | --- | --- | --- | --- | --- | --- | --- | --- | --- | --- | --- | --- | --- | --- | --- | --- | --- | --- | --- | --- | --- | --- | --- | --- | --- | --- | --- | --- | --- | --- | --- | --- | --- | --- | --- | --- | --- | --- | --- | --- | --- | --- | --- | --- | --- | --- | --- | --- | --- | --- | --- | --- | --- | --- | --- | --- | --- | --- | --- | --- | --- | --- | --- | --- | --- | --- | --- | --- | --- | --- | --- | --- | --- | --- | --- | --- | --- | --- | --- | --- | --- | --- | --- | --- | --- | --- | --- | --- | --- | --- | --- | --- | --- | --- | --- | --- | --- | --- | --- | --- | --- | --- | --- | --- | --- | --- | --- | --- | --- | --- | --- | --- | --- | --- | --- | --- | --- | --- | --- | --- | --- | --- | --- | --- | --- | --- | --- | --- | --- | --- | --- | --- | --- | --- | --- | --- | --- | --- | --- | --- | --- | --- | --- | --- | --- | --- | --- | --- | --- | --- | --- | --- | --- | --- | --- | --- | --- | --- | --- | --- | --- | --- | --- | --- | --- | --- | --- | --- | --- | --- | --- | --- | --- | --- | --- | --- | --- | --- | --- | --- | --- | --- | --- | --- | --- | --- | --- | --- | --- | --- | --- | --- | --- | --- | --- | --- | --- | --- | --- | --- | --- | --- | --- | --- | --- | --- | --- | --- | --- | --- | --- | --- | --- | --- | --- | --- | --- | --- | --- | --- | --- | --- | --- | --- | --- | --- | --- | --- | --- | --- | --- | --- | --- | --- | --- | --- | --- | --- | --- | --- | --- | --- | --- | --- | --- | --- | --- | --- | --- | --- | --- | --- | --- | --- | --- | --- | --- | --- | --- | --- | --- | --- | --- | --- | --- | --- | --- | --- | --- | --- | --- | --- | --- | --- | --- | --- | --- | --- | --- | --- | --- | --- | --- | --- | --- | --- | --- | --- | --- | --- | --- | --- | --- | --- | --- | --- | --- | --- | --- | --- | --- | --- | --- | --- | --- | --- | --- | --- | --- | --- | --- | --- | --- | --- | --- | --- | --- | --- | --- | --- | --- | --- | --- | --- | --- | --- | --- | --- | --- | --- | --- | --- | --- | --- | --- | --- | --- | --- | --- | --- | --- | --- | --- | --- | --- | --- | --- | --- | --- | --- | --- | --- | --- | --- | --- | --- | --- | --- | --- | --- | --- | --- | --- | --- | --- | --- | --- | --- | --- | --- | --- | --- | --- | --- | --- | --- | --- | --- | --- | --- | --- | --- | --- | --- | --- | --- | --- | --- | --- | --- | --- | --- | --- | --- | --- | --- | --- | --- | --- | --- | --- | --- | --- | --- | --- | --- | --- | --- | --- | --- | --- | --- | --- | --- | --- | --- | --- | --- | --- | --- | --- | --- | --- | --- | --- | --- | --- | --- | --- | --- | --- | --- | --- | --- | --- | --- | --- | --- | --- | --- | --- | --- | --- | --- | --- | --- | --- | --- | --- | --- | --- | --- | --- | --- | --- | --- | --- | --- | --- | --- | --- | --- | --- | --- | --- | --- | --- | --- | --- | --- | --- | --- | --- | --- | --- |

**Table S3: Detailed overview of neuropathology and donor demographics for cohort 2.**

Tau substrate batch 2 was used (see SFig 1). Tau SAA TTT: Median time to threshold of quadruplicate measures of 10^-6^ brain dilution. HIP: Hippocampus; CER: Cerebellum; Braak Stage (NFTs): Braak staging for Tau neurofibrillary tangles; Braak Stage (LB): Braak staging for Lewy bodies; Amyloid: Deposition of amyloid protein in the brain [[1](#biblioRef00)]; PMD: Post-mortem delay in (h), NBB: Netherlands Brain Bank; AD: Alzheimer’s disease; CTR: Controls; y: years; m: male; f: female; n.a.: not available.

| **# Case** | **Clinical diagnosis** | **Braak  (NFTs)** | **Braak (LB)** | **Amyloid** | **Sex** | **Age (y)** | **PMD (h)** | **Source** | **Region** | **Tau SAA**  **TTT (h)** | **positive replicates**  **(%)** | **Neuropath. Report** |  |
| --- | --- | --- | --- | --- | --- | --- | --- | --- | --- | --- | --- | --- | --- |
|  |  |  |  |  |  |  |  |  |  |  |  |  |  |
| 1 | AD | VI | n.a. | C | m | 84 | 8 | NBB | HIP | 40 | 100 | Many NFTs |  |
|  |  |  |  |  |  |  |  |  | CER | 200 | 0 | No NFTs |  |
| 2 | AD | VI | n.a. | C | f | 72 | 5 | NBB | HIP | 37 | 100 | Many NFTs |  |
|  |  |  |  |  |  |  |  |  | CER | 200 | 0 | n.a. |  |
| 3 | AD | VI | n.a. | C | m | 58 | 5 | NBB | HIP | 25 | 100 | Many NFTs |  |
|  |  |  |  |  |  |  |  |  | CER | 200 | 0 | No NFTs |  |
| 4 | AD | VI | I | C | f | 70 | 5 | NBB | HIP | 200 | 0 | Many NFTs |  |
|  |  |  |  |  |  |  |  |  | CER | 200 | 0 | No NFTs |  |
| 5 | AD | VI | n.a. | C | f | 82 | 5 | NBB | HIP | 37 | 100 | Many NFTs |  |
|  |  |  |  |  |  |  |  |  | CER | 200 | 0 | No NFTs |  |
| 6 | AD | VI | n.a. | C | m | 64 | 4 | NBB | HIP | 33 | 100 | Many NFTs |  |
|  |  |  |  |  |  |  |  |  | CER | 200 | 0 | No NFTs |  |
| 7 | AD | V | n.a. | C | f | 74 | 6 | NBB | HIP | 27 | 100 | Many NFTs |  |
| 8 | AD | V | n.a. | C | m | 77 | 5 | NBB | HIP | 26 | 100 | Many NFTs |  |
| 9 | AD | V | n.a. | C | f | 79 | 4 | NBB | HIP | 33 | 100 | Many NFTs |  |
| 10 | AD | V | n.a. | C | m | 61 | 4 | NBB | HIP | 39 | 100 | Few NFTs |  |
| 11 | AD | VI | n.a. | C | f | 70 | 6 | NBB | HIP | 32 | 100 | Many NFTs |  |
| 12 | Non-demented CTR | II | 0 | 0 | f | 93 | 7 | NBB | HIP | 200 | 0 | No NFTs |  |
|  |  |  |  |  |  |  |  |  | CER | 200 | 0 | No NFTs |  |
| 13 | CTR with cerebrovascular accident | II | n.a. | A | f | 85 | 7 | NBB | HIP | 101 | 100 | Few NFTs |  |
|  |  |  |  |  |  |  |  |  | CER | 200 | 0 | n.a. |  |
| 14 | Non-demented CTR with Lewy bodies | I | III | A | f | 92 | 7 | NBB | HIP | 83 | 100 | Few  NFTs |  |
|  |  |  |  |  |  |  |  |  | CER | 200 | 0 | n.a. |  |
| 15 | Non-demented CTR with Lewy bodies | II | III | A | f | 95 | 5 | NBB | HIP | 71 | 100 | Moderate NFTs |  |
|  |  |  |  |  |  |  |  |  | CER | 200 | 0 | No NFTs |  |
| 16 | Epilepsy | I | n.a. | O | m | 86 | 11 | NBB | HIP | 200 | 0 | No NFTs |  |
|  |  |  |  |  |  |  |  |  | CER | 200 | 0 | n.a. |  |
| 17 | Epilepsy | I | n.a. | A | f | 86 | 8 | NBB | HIP | 90 | 100 | Few  NFTs |  |
|  |  |  |  |  |  |  |  |  | CER | 200 | 0 | no NFT |  |
| 18 | Non-demented CTR with Lewy bodies | I | n.a. | B | m | 84 | 7 | NBB | HIP | 79 | 100 | Few NFTs |  |
|  |  |  |  |  |  |  |  |  | CER | 200 | 0 | no NFT |  |
| 19 | Non-demented CTR with Lewy bodies | I | V | C | m | 75 | 6 | NBB | HIP | 67 | 100 | Many -few NFTs |  |
|  |  |  |  |  |  |  |  |  | CER | 200 | 0 | No NFTs |  |
| 20 | CTR with cerebrovascular accident | I | III | O | m | 70 | 6 | NBB | HIP | 169 | 100 | No NFTs |  |
| 21 | Non-demented CTR with Lewy bodies | I | n.a. | n.a. | f | 91 | 4 | NBB | HIP | 70 | 100 | Few NFTs |  |

**Table S4:** **Detailed** **overview of neuropathology and donor demographics for cohort 3**.

Tau substrate batch 1 was used. Tau SAA TTT: Median of triplicated measures of 10^-5^ brain dilution. Braak Stage (NFTs): Braak staging for Tau neurofibrillary tangles; Braak Stage (LB): Braak staging for Lewy bodies; Amyloid: Deposition of amyloid protein in the brain, amyloid neuropathological classification described in [[1](#biblioRef00)]; PMD: Post mortem delay; NBB: Netherlands Brain Bank; MFG: Middle frontal Gyrus; y: years; m: male; f: female; n.a.: not available.

| **# Case** | **Neuropathological**  **diagnosis** | **Braak (NFTs)** | **Braak stage**  **(Lewy Bodies)** | **Amyloid deposition [1]** | **Sex** | **Age**  **(y)** | **PMD**  **(h)** | **Source** | **Region** | **Tau SAA**  **TTT (h)**  **10^-5^** | **positive replicates**  **(%)**  **10^-5^** | **IHC**  **AT100** |
| --- | --- | --- | --- | --- | --- | --- | --- | --- | --- | --- | --- | --- |
| 1 | Non-demented CTR | 0 | n.a. | A | m | 93 | 7 | NBB | MFG | 430 | 0 | 0 |
| 2 | Non-demented CTR | I | 0 | B | f | 85 | 7 | NBB | MFG | 430 | 0 | 0 |
| 3 | Non-demented CTR | I | 0 | A | f | 78 | 7 | NBB | MFG | 430 | 0 | n.a. |
| 4 | Non-demented CTR | I | 0 | A | f | 89 | 13 | NBB | MFG | 430 | 0 | 0 |
| 5 | Non-demented CTR | II | n.a. | n.a. | f | 97 | 10 | NBB | MFG | 135 | 100 | n.a. |
| 6 | Non-demented CTR | II | 0 | A | m | 82 | 5 | NBB | MFG | 415 | 33 | n.a. |
| 7 | Non-demented CTR | II | n.a. | C | m | 80 | 4 | NBB | MFG | 134 | 100 | ++ |
| 8 | Non-demented CTR | II | n.a. | O | m | 90 | 5 | NBB | MFG | 430 | 0 | 0 |
| 9 | Non-demented CTR | II | 0 | n.a. | f | 88 | 6 | NBB | MFG | 430 | 0 | n.a. |
| 10 | Non-demented CTR | II | n.a. | n.a. | f | 76 | 4 | NBB | MFG | 348 | 67 | 0 |
| 11 | Non-demented CTR | II | n.a. | C | f | 99 | 4 | NBB | MFG | 141 | 100 | n.a. |
| 12 | Non-demented CTR | III | 0 | B | f | 80 | 7 | NBB | MFG | 228 | 100 | ++ |
| 13 | Non-demented CTR | III | n.a. | B | f | 95 | 7 | NBB | MFG | 430 | 0 | 0 |
| 14 | Non-demented CTR | III | n.a. | n.a. | f | 89 | 6 | NBB | MFG | 353 | 33 | 0 |
| 15 | Non-demented CTR | III | 0 | A | f | 88 | 5 | NBB | MFG | 378 | 67 | 0 |
| 16 | Non-demented CTR | III | 0 | A | f | 81 | 5 | NBB | MFG | 347 | 33 | 0 |
| 17 | Non-demented CTR | III | n.a. | A | f | 85 | 6 | NBB | MFG | 239 | 67 | + |
| 18 | Non-demented CTR | III | n.a. | A | f | 85 | 8 | NBB | MFG | 92 | 100 | 0 |
| 19 | AD | IV | 0 | C | f | 91 | 6 | NBB | MFG | 74 | 100 | ++ |
| 20 | AD | IV | n.a. | B | f | 96 | 7 | NBB | MFG | 430 | 0 | 0 |
| 21 | AD | IV | n.a. | C | m | 79 | 4 | NBB | MFG | 152 | 100 | + |
| 22 | AD | IV | n.a. | C | f | 100 | 5 | NBB | MFG | 113 | 100 | ++ |
| 23 | AD | IV | n.a. | C | f | 72 | 6 | NBB | MFG | 154 | 100 | +++ |
| 24 | AD | IV | n.a. | C | f | 82 | 5 | NBB | MFG | 190 | 100 | + |
| 25 | AD | IV | n.a. | n.a. | m | 89 | 4 | NBB | MFG | 430 | 0 | + |
| 26 | AD | IV | n.a. | C | f | 94 | 8 | NBB | MFG | 96 | 100 | + |
| 27 | AD | IV | n.a. | C | f | 81 | 6 | NBB | MFG | 121 | 100 | ++/+ |
| 28 | AD | IV | n.a. | B | m | 91 | 4 | NBB | MFG | 187 | 100 | ++ |
| 29 | AD | V | n.a. | C | m | 78 | 6 | NBB | MFG | 157 | 100 | +++ |
| 30 | AD | V | n.a. | C | m | 82 | 5 | NBB | MFG | 70 | 100 | +++ |
| 31 | AD | V | n.a. | C | f | 71 | 4 | NBB | MFG | 86 | 100 | +++ |
| 32 | AD | V | n.a. | C | f | 70 | 5 | NBB | MFG | 95 | 100 | +++ |
| 33 | AD | V | n.a. | C | m | 74 | 8 | NBB | MFG | 96 | 100 | +++ |
| 34 | AD | V | n.a. | C | f | 73 | 4 | NBB | MFG | 63 | 100 | +++ |
| 35 | AD | VI | n.a. | C | m | 64 | 4 | NBB | MFG | 87 | 100 | +++ |
| 36 | AD | VI | n.a. | C | f | 71 | 3 | NBB | MFG | 59 | 100 | +++ |
| 37 | AD | VI | I | C | f | 64 | 5 | NBB | MFG | 48 | 100 | +++ |
| 38 | AD | VI | n.a. | C | f | 84 | 4 | NBB | MFG | 73 | 100 | ++ |
| 39 | AD | VI | n.a. | C | f | 82 | 5 | NBB | MFG | 60 | 100 | +++ |
| 40 | AD | VI | n.a. | C | m | 55 | 4 | NBB | MFG | 74 | 100 | +++ |
