# Supplementary material for: Correction: Tau seed amplification assay reveals relationship between seeding and pathological forms of tau in Alzheimer’s disease brain
Source: Acta Neuropathol Commun. 2024 Jul 15;12:115. doi: 10.1186/s40478-024-01825-9 (PMC11247772; doi:10.1186/s40478-024-01825-9)
Supplement: Supplementary file 1 — Supplementary Material 1 [file 40478_2024_1825_MOESM1_ESM.docx]

**SUPPLEMENTARY TABLES**

**Table S1: Summary of Tau SAA substrates tested with Alzheimer’s disease (AD) and control (CTR) brain homogenate.** TTT: time to threshold value from Tau SAA based brains diluted by 10^-5^.

| **Category** | **Source** | **Tau isoform** | **Mutations** | **Tag** | **Self-aggregation** "-" = no aggregation "+" = aggregation | **Tau SAA results: ratio TTT (CTR) / TTT (AD))** "-" = no difference "+" = 1.1-1.5-fold difference  "+++" = >2.5-fold difference |
| --- | --- | --- | --- | --- | --- | --- |
| Full-length 4R | In-house | 2N4R |  | untagged | - | - |
|  | In-house | 2N4R | C291A, C322A | untagged | - | - |
|  | In-house | 2N4R | ∆K280, C291A, C322A | untagged | - | - |
|  | In-house | 0N4R |  | untagged | - | - |
|  | Senostic Health GmbH | 0N4R | C291S, C322S | N-terminal His6-tag | + | + |
| Full-length 3R | In-house | 2N3R | C322A | untagged | - | - |
|  | In-house | 0N3R |  | untagged | - | - |
|  | In-house | 0N3R | C322S | untagged | - | - |
|  | Senostic Health GmbH | 0N3R | C322S | N-terminal His6-tag | - | +++ |
| Full-length 4R/3R ratio 1:1 | In-house | 2N4R 2N3R | C291A, C322A C322A | untagged | - | - |
|  | Senostic Health GmbH | 0N4R 0N3R | C291S, C322S C322S | N-terminal His6-tag N-terminal His6-tag | + | + |
